# Genomic Insight into *Pediococcus acidilactici* HN9, a Potential Probiotic Strain Isolated from the Traditional Thai-Style Fermented Beef Nhang

**DOI:** 10.3390/microorganisms9010050

**Published:** 2020-12-27

**Authors:** Komwit Surachat, Duangporn Kantachote, Panchalika Deachamag, Monwadee Wonglapsuwan

**Affiliations:** 1 Division of Computational Science, Faculty of Science, Prince of Songkla University, Hatyai, Songkhla 90110, Thailand; 2 Molecular Evolution and Computational Biology Research Unit, Faculty of Science, Prince of Songkla University, Hatyai, Songkhla 90110, Thailand; 3 Division of Biological Science, Faculty of Science, Prince of Songkla University, Hatyai, Songkhla 90110, Thailand; duangporn.k@psu.ac.th (D.K.); passanee.d@psu.ac.th (P.D.); monwadee.wo@psu.ac.th (M.W.)

**Keywords:** beneficial lactic acid bacteria, pediococci, *Pediococcus acidilactici*, comparative genome analysis, fermented food

## Abstract

*Pediococcus acidilactici* HN9 is a beneficial lactic acid bacterium isolated from Nhang, a traditional Thai-style fermented beef. In this study, the molecular properties of *P. acidilactici* HN9 were characterized to provide insights into its potential probiotic activity. Specifically, this work sought to report the complete genome of *P. acidilactici* HN9 and perform a comparative genome analysis with other bacterial strains belonging to the genus *Pediococcus*. Genomic features of HN9 were compared with those of all other bacterial *Pediococcus* strains to examine the adaptation, evolutionary relationships, and diversity within this genus. Additionally, several bioinformatic approaches were used to investigate phylogenetic relationships, genome stability, virulence factors, bacteriocin production, and antimicrobial resistance genes of the HN9 strain, as well as to ensure its safety as a potential starter culture in food applications. A 2,034,522 bp circular chromosome and two circular plasmids, designated pHN9-1 (42,239-bp) and pHN9-2 (30,711-bp), were detected, and used for pan-genome analysis, as well as for identification of bacteriocin-encoding genes in 129 strains belonging to all *Pediococcus* species. Two CRISPR regions were identified in *P. acidilactici* HN9, including type II-A CRISPR/CRISPR-associated (Cas). This study provides an in-depth analysis on *P. acidilactici* HN9, facilitating a better understanding of its adaptability to different environments and its mechanism to maintain genome stability over time.

## 1. Introduction

*Pediococcus*, a genus of gram-positive lactic acid bacteria (LAB), is classified as part of the *Lactobacillaceae* family [1]. These bacteria, which are commonly found in fermented vegetables, meats, and dairy products, play an important physiological role in fermentation [2,3,4]. Several species of *Pediococcus* are used in the food industry, owing to their inhibitory effects on other microorganisms via antimicrobial peptide production [5,6]. In addition, there are many reports of bacteriocins produced by *Pediococcus* strains, including Pediocin F [7], Pediocin PA-1 [8], and Pediocin SA-1 [9]. *Pediococcus acidilactici* is a potent producer of bacteriocins that has been used in the food industry for many years [3,4,10,11]. Moreover, several studies have reported that *P. acidilactici* can inhibit the growth of pathogens during the fermentation process and food storage [4,6,12,13].

*Pediococcus acidilactici* strains have been widely studied in food and feed applications. For instance, *P. acidilactici* SMVDUDB2 [14] was found to be capable of phytate degradation and to possess probiotic traits, which can be useful in food and feed applications. Interestingly, probiotic *P*. *acidilactici* MA18/5M reportedly modulates antiviral responses in Atlantic salmon when used as a dietary supplement [15], while strain CNCM I-4622 (trade name: Bactocell^®^) has been authorized by The Panel on Additives and Products or Substances used in Animal Feed (FEEDAP) as a feed additive for all fish, shrimps, and crustaceans [16]. Additionally, *P. acidilactici* R037 has been reported to induce interleukin (IL)-10-producing regulatory T cells and to have demonstrated efficacy in treating multiple sclerosis via oral administration in mice [17].

It has long been known that the use of starter culture in fermented foods in order to improve the quality and produce safe food compared with spontaneous fermentation. *P. acidilactici* strains have key enzymes involved in homo-fermentation including D- and L- lactate dehydrogenases [14]. Therefore, they can quickly produce lactic acid to reduce fermentation time. Consequently, *P. acidilactici* strains are used generally as the starter culture in fermented meat products including *P. acidilactici* ATCC 8042 [18], *P. acidilactici* MCH14 [19] since they can grow and survive at different temperatures, salt concentrations, and pH values [20,21,22]. These physiological traits contribute and play an important role in beef fermentation. Also, these strains can inhibit the growth of the foodborne pathogens *Listeria monocytogenes* and *Clostridium perfringens* [19]. Additionally, to gain more benefits from starter culture, its probiotic properties should be considered by testing its property to survive in the gastrointestinal tract for promoting health benefits to the host. Based on this property, LAB including *P. acidilactici* strains are potential candidates to be probiotics, as their resistance in acidity. Hence, fermented foods, e.g. dairy products, and fermented meat products are important probiotic sources for humans and animals.

However, only a few studies were focused on the comparative genomics of *P. acidilactici* strains. For example, Ranjan et al. [23] proposed the genome sequence of *P. acidilactici* NRCC1 isolated from dromedary camel to compare genomic features with reference strain (*P. acidilactici* DSM 20284). Also, Snauwaert et al. [24] presented the draft genome of *P. damnosus* LMG 28219 and performed a comparative analysis against several genomes, including *P. acidilactici,* to underly the mechanisms of its adaptation to the beer niche. In particular, pan-genome analysis for the exploration of genus and species diversity, as well as the ability of these bacteria to produce bacteriocins, is required to gain a comprehensive understanding of these different aspects, including their associated safety when present in food products.

In current practice, before the application of any potent microbial strain as a starter culture for producing fermented food/beverage, its safety profile must be investigated—particularly drug resistance genes and virulence factors. To date, performing whole genome analyses presents the most effective method for evaluating the safety of any strain. This approach also provides insights into the presence of beneficial genes that encode bacteriocins and oligopeptides, etc., which may offer additional value to the fermented food, allowing them to serve as functional food. Therefore, the aims of this study were to determine the complete genome of *P. acidilactici* HN9 isolated from the traditional Thai-style fermented beef Nhang and to perform comparative genomic analysis of all bacterial strains in the genus *Pediococcus*. Furthermore, to display the diversity within this genus, the relationships among all strains were analyzed using the phylogenetic tree generated with all core genes in this genus. Specifically, we used 129 assemblies downloaded from the NCBI database to perform pan-genome analysis. This is the first study, to our knowledge, to provide a meta-analysis for all bacterial strains in the genus *Pediococcus* and to identify bacteriocins produced. In addition, the safety of using *P. acidilactici* HN9 in food and beverage applications was evaluated using genomic data.

## 2. Materials and Methods 

### 2.1. Bacterial Strain and DNA Extraction

*Pediococcus acidilactici* HN9 was isolated from fermented beef using the de Man, Rogosa, and Sharp (MRS, Merck, Darmstadt, Germany) broth at the Faculty of Science, Prince of Songkla University. This strain was selected based on its antibacterial activity against foodborne pathogens and usability as a starter culture for producing fermented meat products. A single colony of *P. acidilactici* HN9 was cultivated in the MRS broth leaving a headspace in the culture tube to achieve a microaerophilic condition at 37 °C for 24 h. Genomic DNA was then extracted and purified using DNeasy extraction kit (QIAGEN, Hilden, Germany) following the manufacturer’s instructions as described previously [25]. 

### 2.2. Whole Genome Library Preparation and Sequencing

Two whole genome libraries were prepared to perform both short-read and long-read sequencing. High-quality genomic DNA of *P. acidilactici* HN9 was extracted for use in both libraries. For long-read sequencing, purified genomic DNA was sequenced with a PacBio RSII sequencer (Pacific Biosciences, Menlo Park, CA, USA). After constructing the RSII library, sequences were generated using P4-C2 chemistry in a single-molecule real-time (SMRT) cell [26], yielding 139,184 subreads with an average read length of approximately 4.6 kbp. Short-read sequencing was performed in-house at Prince of Songkla University using a NextSeq 550 sequencer (Illumina, Inc., San Diego, CA, USA). Libraries were prepared using Nextera XT DNA Library Prep Kit (Illumina) according to the manufacturer’s instructions. All reads generated by the sequencer were 150-bp paired-end reads.

### 2.3. Genome Assembly

SMRTbell adapters and short polymerase reads were removed from the raw reads of PacBio sequencing, followed by removal of low-quality reads (lower than 0.85) and short subreads (<1000 bp) using Canu [27] with -correct parameter to replace noisy read sequences that had representative sequences computed on the basis of overall overlapping reads. The raw reads were trimmed with -trim parameter to select high-quality sequences for further analysis. We obtained 126,932 raw reads from the PacBio sequencer, with a total yield of 771 Mbp. The initial draft of the genome assembly was generated using the -assemble parameter with the genome size set at 2.6 Mbp. The draft assembly was circularized using Circlator [28] to identify and trim the overhangs on chromosomes and plasmids using the corrected reads and the draft assembly obtained using Canu software. 

### 2.4. Small Plasmid Identification and Assembly

PacBio reads are long and may exceed the lengths of small plasmid sequences with a size smaller than 10 kb [29]. During assembly, a parameter setting on the seed read length cut-off that is greater than actual plasmid size in the hierarchical genome assembly process (HGAP) software [30] may miss small plasmids [31]. In order to scan a small plasmid, all short-reads from NextSeq 550 sequencer were aligned to the draft assembly using BWA-mem [32] and the unmapped Illumina reads were extracted from the alignment file (.bam) using SAMtools [33]. The unmapped reads were assembled with Spades version 3.13.0 [34]. We only selected contigs with high coverage to perform trimming and fix the orientation of the plasmids. A blast database was created using the high coverage contigs with makeblastdb command in the NCBI-blast suite program to perform trimming of small plasmids [35]. To ensure that the selected sequences were indeed the plasmid, the PlasmidFinder v. 2.1 [36] was used to scan and exclude redundant sequences including ribosomal DNA or transposons from analysis.

Then, the initial 5 lines of the fasta sequences of the contigs were extracted for searching against all assemblies. If the search returned more than two hits (the first hit should come from the start of the sequence), the overhang of the plasmid could be identified. The overhanging bases were trimmed at the position before the start of the second hit. Finally, the plasmid contig orientation was rearranged. The bacterial chromosome was oriented at the gene encoding DnaA, while plasmids were usually oriented at the replication gene [37]. The Circlator tool was then used to fix the orientation of the plasmid using the fixstart tool. Thus, complete plasmid sequences were finally obtained. 

### 2.5. Assembly Polishing and Evaluation

The assembly was polished to correct the draft assembly of long-read sequences using short-read sequences. First, an alignment file was generated using Illumina paired-end reads to align back to the draft PacBio assembly generated using BWA-mem. Then, Pilon [38], a software for correcting incorrect bases, was applied to polish the sequences of the chromosome and all plasmids of *P. acidilactici* HN9. Finally, the quality of the assembly was evaluated using Busco software version 4.1.2 [39,40]. Busco can provide information regarding annotation completeness based on evolutionarily informed expectations of gene content. 

### 2.6. Genome Annotation and Visualization

Gene finding was performed using Glimmer 3.0 [41]. Functional annotations were predicted with Rapid Annotations using the Subsystems Technology (RAST) [42,43,44] and the NCBI Prokaryotic Genome Annotation Pipeline [45] in our local server. We identified all tRNAs and rRNAs with tRNAscan-SE [46] and RNAmmer [47], respectively. The clustered regularly interspaced short palindromic repeats (CRISPRs) and tandem repeats were identified using CRISPRFinder [48] and Tandem Repeat Finder [49], respectively. Finally, the genomic content was visualized with the CGView server [50,51,52].

### 2.7. Comparative Genomic Analysis

All 129 genome assemblies of the genus *Pediococcus* were compared, and pan-genome analysis was performed, including all complete genome, scaffold, and contig level sequences. Further, we performed a pan-genome analysis at the species level of *P. acidilactici* with 16 complete genome, 11 scaffold, and 21 contig level sequences of 54 strains from the GenBank database (accessed date: 3 September 2020). The assembly details and accession numbers are illustrated in Supplementary Table S1 and S2. The annotation files of all assemblies downloaded from the GenBank database were regenerated with the same protocol to reduce biases arising due to different gene calling methods. Prokka version 1.14.641 [53] was used to annotate all assemblies for creating the General Feature Format (GFF) files. The pan-genome analyses were performed using Roary version 3.13.0 [54] with a minimum BLASTp identity of 60% and a threshold of 97% of isolates a gene must be in to be defined as a conserved gene. Two analyses for pan-genome were identified by retrieving the group of genes shared by all strains in the clade of interest (core genome) and the group of genes that are not present in all the strains (accessory genome). The pan-genome is a combination of all the genes that are found in the clade of interest. Therefore, we first compared the genome assemblies of all strains in the genus *Pediococcus* (129 strains). Second, we used only strains at the species level (45 strains) to compute the pan-genome. All related genes, including core, accessory, and unique gene sets, were queried using Roary scripts.

### 2.8. Phylogenetic Analyses

The single-copy marker genes computed using Roary were concatenated and aligned using MUSCLE [55]. A phylogenetic tree was then constructed using RaxMLHPC [56] with the neighbor-joining method. The number of bootstrap repetitions was set to 500 to test tree reliability. Tree visualization was illustrated in Geneious R10.2.6 software (Biomatters Ltd., Auckland, New Zealand).

### 2.9. Multiple Genome Alignments Analysis

To further analyze the evolutionary relationships among the species, we selected the complete genomes in the same clade of the phylogenetic tree to construct alignments using the MAUVE [57] plugin in Geneious software [58].

### 2.10. Identification of Bacteriocin-Encoding Genes

Bacteriocin-encoding genes and gene clusters were predicted and illustrated using Bagel4 web server [59,60]. To determine the presence of bacteriocin operons in all strains used in this study, BLASTp was performed against the BAGEL database [60].

### 2.11. Identification of Carbohydrate-Active enZyme (CAZyme)

Genes associated with Carbohydrate-active enzymes (CAZymes) families were identified by searching against the CAZy database [61]. All protein sequences annotated from the genome annotation process were scanning using the dbCAN server [62] with HMMER v. 3.3.2 [63] against CAZy database. An E-value of 1e-15 and coverage of 0.35 were set as the cutoff threshold for identifying CAZyme Class [64]. 

### 2.12. In Silico Comparison of Genes Associated with Meat Fermentation

Genes associated with meat fermentation including *asnA2, pgm, uppS, asnS, tnpA1, ctsR, ldhL*, *ldhD, ctsR* and *rrp-3,* were downloaded from the NCBI server to create a local database for performing BLAST analysis. Then, every genome was then searched against the created database using BLASTn with an E-value cutoff at 1e-15 to determine the presence/absence of the gene. The visualization of the result was presented in the heatmap using R script.

### 2.13. Identification of Antibiotic Resistance Genes and Virulence Factors

Pathogenic genes, antibiotic resistance genes, and virulence factors were identified to assess the safety of *P. acidilactici* HN9 use. The genomic sequences were searched against several databases, including the Comprehensive Antibiotic Resistance Database (CARD) [65], ResFinder [66] and Virulence Factors Database (VFDB) [67]. Resistance Gene Identifier (RGI) was used against the CARD database to predict resistomes from proteins based on homology and SNP models [65]. The criteria of RGI identification were classified into three types: “Perfect hit” (100% identical with reference), “String hit” (Bit score > 450 and not identical), and “Loose hit” (Bit score < 450, matched in some regions). In addition, ResFinder 4.0 was used to scan for antimicrobial resistance (AMR) genes with a 90% identity threshold and 60% minimum length. The probability of being a human pathogen was determined using the PathogenFinder [68]. Additionally, CRISPRs, prophage regions, and insertion sequences were predicted using CRISPRFinder [48], PHASTER [69], and ISfinder [70], respectively.

## 3. Results

### 3.1. General Genome Characteristics and Functional Annotation of P. acidilactici HN9

**Figure 1:**
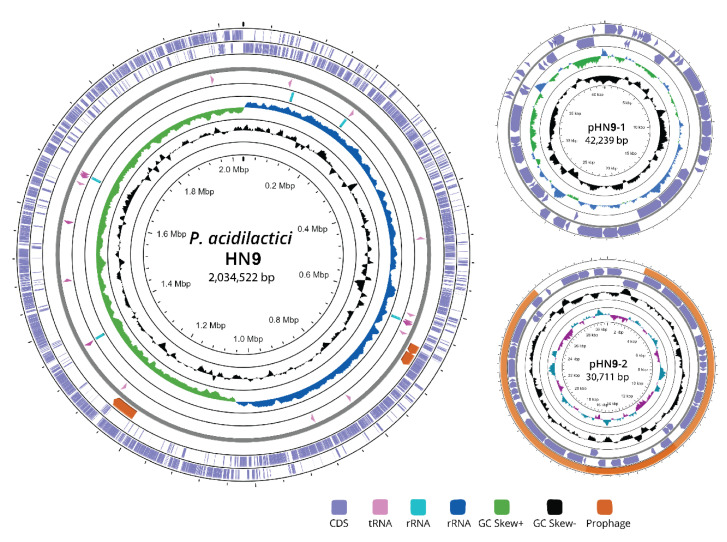
Circular genome and plasmid representations of *P. acidilactici* HN9. The circular illustrations were visualized using the CGView server beta version and contain seven rings. The outermost circle and the second circle show the positions of the CDSs in forward and reverse strand directions, respectively. The next rings show the following information: prophage regions, tRNA, rRNA, GC content, GC skew+, and GC skew-, respectively.

The genome of *P. acidilactici* HN9 comprises one 2,034,522-bp circular chromosome and two circular plasmids, designated as pHN9-1 (42,239-bp) and pHN9-2 (30,711-bp). The circular representations of *P. acidilactici* HN9 chromosome and plasmids are visualized as shown in Figure 1. General information of *P. acidilactici* HN9 genome and its genomic features, including prophages, CRISPR region, and AMR gene, are given in Table 1. For functional annotation by RAST subsystems, we identified 2013 coding sequences assigned in 200 subsystems in the chromosome. Only 590 (29%) of all coding sequences (CDS) were hypothetical or unknown. Most assigned subsystems were Carbohydrates (169); Protein Metabolism (119); and Cofactors, Vitamins, Prosthetic Groups, and Pigments (66), respectively. Interestingly, 12 CDS were identified in the Fermentation subcategory including lactate (8), as well as acetoin and butanediol metabolism (4). Also, we found 5 CDS of Restriction-Modification (R-M) System including *HsdR*, *HsdM*, and *HsdS*.

In the case of the plasmids, a total of 54 and 42 CDS were identified in pHN9-1 and pHN9-2, respectively. In pHN9-1, most of the CDS identified were hypothetical proteins (11), mobile element proteins (8), transposases (6), and oligopeptide ABC transporters (5). These CDS were assigned into only two subsystems: Stress Response (1) and Carbohydrates (3) by RAST server. In pHN9-2, most of the CDS identified were hypothetical proteins (15), mobile element proteins (2), plasmid replication initiation protein (2) and sucrose permease (2). Also, only two subsystems were predicted in pHN9-2; these were Sulfur Metabolism (2) and Carbohydrates (6). Detailed information on the subsystems is illustrated in Figure S1. In addition, Microbial Nucleotide BLAST analysis against bacterial database was performed in the NCBI server to find closely associated genomes. As expected, the top-five hits of the pHN9-1 search belonged to plasmids of *P. acidilactici* with 99% identity and 70% coverage; these were *P. acidilactici* SRCM102732 (NZ_CP028250), SRCM102731 (NZ_CP028248), SRCM100313 (NZ_CP021488.1), SRCM100424 (NZ_CP021485), and SRCM101189 (NZ_CP021530), respectively. The sequence of pHN9-1 had higher similarities to plasmids of *P. pentosaceus* ATCC 25745 (NC_008525) and *Lactiplantibacillus plantarum* ATCC 8014 (NZ_CP024413) with identities over 99%, although coverages of the sequences were only 30%. BLAST identification of pHN9-2 revealed that the sequence was more similar to that of the *Lactiplantibacillus* group, with over 99% identity and 30% coverage.

### 3.2. Core and Pan-Genomes of P. acidilactici Strains

**Figure 2:**
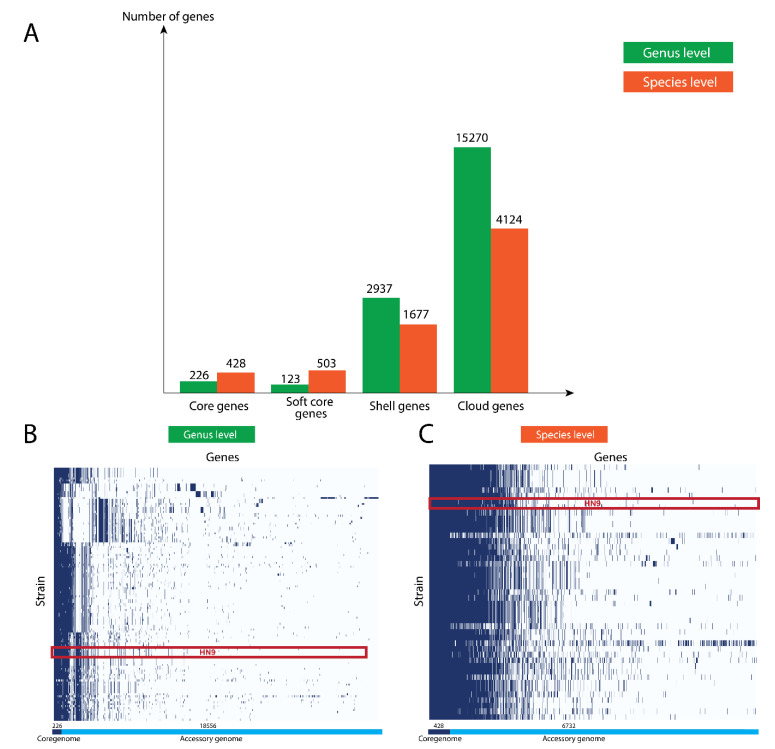
Pan-genome analyses of *P. acidilactici* HN9 at species and genus levels determined using Roary software. 45 and 129 strains were compared at the species and genus levels, respectively. (**A**) The summary of pan-genome analyses at both levels. (**B**,**C**) plots of pan-genome matrices-based genus and species levels, respectively. The presence of each gene in each strain is indicated by the blue color, while the white color indicates the absence of the gene from the strain. The red box represents a set of genes of *P. acidilactici* HN9.

We searched the complete genome of *P. acidilactici* HN9 against the microbial database in the NCBI server. The hits from the BLAST search with the highest similarities had 99% identity and 90% coverage. The strains sharing the highest similarities were *P. acidilactici* SRCM103367, SRCM103444, and JQII-5. The genome diversity among related bacterial strains can be evaluated using pan-genome analysis. Interestingly, we found the ratios of core genes, soft-core genes, shell genes, and cloud genes at the genus versus the species level to be (226: 428), (123: 503), (2937: 1677), and (15,270: 4124), respectively, as shown in Figure 2. Core genes were found in >99% of the genomes, soft core genes were found in 95 to 99%, shell genes were found in 15 to 95%, and cloud genes were found in <15%.

### 3.3. Unique Genetic Content of HN9

The proportion of strain-specific genes found only in HN9 was around 6.16%. Among the 124 unique genes, 31 and 93 encoded proteins identified with known functions and hypothetical proteins, respectively. Strain HN9 carried several genes involved in the transport of sucrose, including *cscB_1* and *cscB_2*. These genes are related to the transport of maltose, fructose, and lactulose into the cell [71]. Further, we detected Sucrose-6-phosphate hydrolase (*sacA_1* and *sacA_2*) with 100% coverage and 95% identity of amino acid sequence to the reference sequence (WP_173819270), which enables this strain to metabolize sucrose as the sole carbon source. The other remaining genes were insertion sequence elements, IS30 family transposase ISLpl1, and IS3 family transposase IS1223, which are commonly found in the *Lactobacillus* group and other lactic acid bacteria. These genes may have been transferred among lactic acid bacteria during their evolution.

### 3.4. Phylogenetic Analysis and Comparison

**Figure 3:**
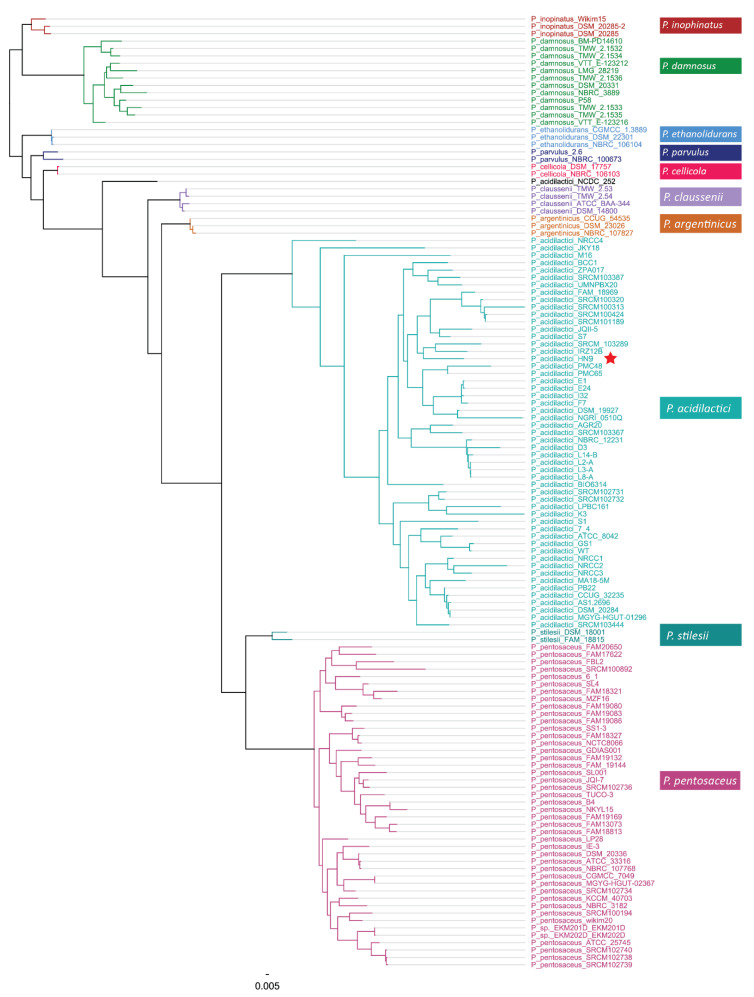
Phylogenetic analysis of *Pediococcus acidilactici* HN9 and other strains at the genus level, based on 33,568 pan genes from 129 strains. The tree was constructed using the neighbor-joining method with a bootstrap of 500 replications by concatenating the single-copy marker genes computed using Roary. The scale bar represents an evolutionary distance. The red star represents the location of *P. acidilactici HN9*.

The phylogenetic trees were constructed based on the pan and core genes (Figure 3 and Figure S2) at the genus level, with a total of 129 strains obtained from the NCBI database. Figure 3 shows that *P. acidilactici* HN9 is grouped together with *P. acidilactici* members and explicitly separated from other groups. However, the genus level phylogenetic tree could not distinguish among the members of the *P. acidilactici* group. Therefore, we constructed another phylogenetic tree based on the core genes of all strains found in the *P. acidilactici* group to further analyze the phylogenetic relationship among the *P. acidilactici* strains. The tree was built based on 426 core genes sharing a similarity of over 60% of amino acid sequences, as shown in Figure S3. HN9 showed the closest relationship with IRZ12B, with the bootstrap value of each node being 100 in this clade. IRZ12B was also isolated from food in Iran under BioSample number SAMN15337232. IRZ12B and HN9 shared 1653 core genes (85%) and contained the bacteriocin-encoding gene, Enterolysin A. IRZ12B contained over 300 unique genes compared to HN9, from which 195 were identified as uncharacterized proteins. Moreover, HN9 was close to other strains, including JQII-5, S7, SRCM100313, SRCM100320, SRCM101189, and SRCM100424, which formed the nearest clade.

### 3.5. Multiple Genome Alignments Analysis

**Figure 4:**
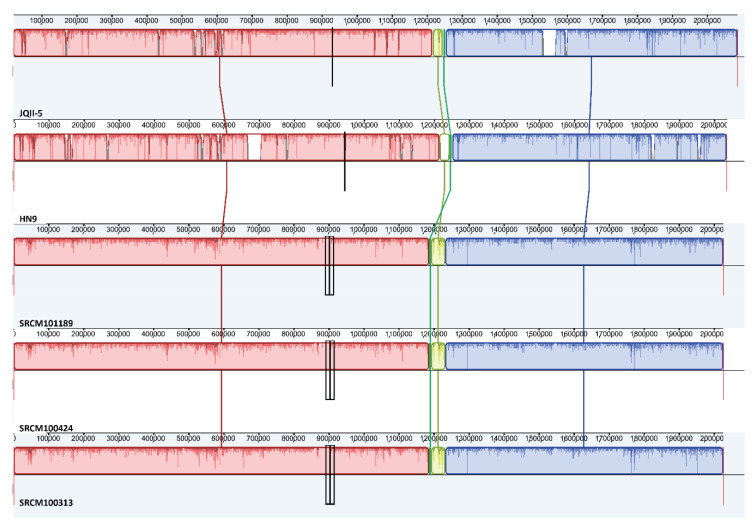
Multiple genome alignment of *Pediococcus acidilactici* strains, including HN9, JQII-5, SRCM100313, SRCM101189, and SRCM100424. Visualization of alignment is arranged into one horizontal panel per genome sequence, with the label of the genome sequence name on the bottom-left of each panel. The homologous blocks are represented with the same color and are connected within the genome by lines.

Multiple genome alignments represent evolutionary changes in the nucleotides by aligning homologous regions of sequences [57]. Therefore, four genomes that were closest to that of HN9 were selected based on their locations in the phylogenetic tree and the level of completeness of their assembly, as shown in Figure 4. Only complete genomes were selected for multiple genome alignments; the following strains were included: JQII-5, SRCM100313, SRCM101189, and SRCM100424. Multiple genome alignments showed that the five genomes shared three main regions Strain JQII-5, SRCM100313, SRCM101189, and SRCM100424, shared certain similarities. However, strain HN9 was different from these strains, as shown in the lime colored region of Figure 4; the region of similarity among the four other strains was replaced by various phage proteins, such as phage major capsid protein, phage portal, and phage terminase, in HN9. This illustrates how this strain diverges from the other strains and provides important evidence of HN9 reconstruction during its evolution over time.

### 3.6. Genome Stability

To evaluate genome stability, we investigated the presence of prophages, mobile genetic elements, and insertion elements. In HN9, we found three prophage regions predicted by the PHASTER server, including one incomplete and two intact prophage regions. The information of each region is given in Table 2. Most of the components in every phage region are hypothetical proteins and phage-related proteins from various genera, including *Lactobacillus*, *Streptococcus,* and *Bacillus*. 

Insertion sequences (IS) in HN9 were identified using ISfinder. Most IS originated in the genera *Lactobacillus* (*L. plantarum*, *L. fructivorans*, *L. johnsonii*, and *L. sakei*), and *Pediococcus* (*P. pentosaceus*), including ISLpl1, ISPp1, ISLfr1, ISLjo2, and ISLjo2. We also found IS from the species *Enterococcus hirae* (IS1310), *Bacillus megaterium* (ISBame1), *Leuconostoc mesenteroides* (IS1165), and *Weissella cibaria* (ISWci1), revealing the mechanism of HN9 adaptation for survival via integration of these elements to its genome. In addition, two CRISPR loci were identified in the genome of *P. acidilactici* HN9, including type II-A of CRISPR/CRISPR-associated (Cas). We found two candidates of the CRISPR region (1 confirmed, 1 questionable) in the HN9 genome, which belonged to the type II-A CRISPR/CRISPR-associated (Cas) system that includes *cas1, cas2, cas9, and csn2*.

### 3.7. Identification of Antimicrobial Peptides

**Figure 5:**
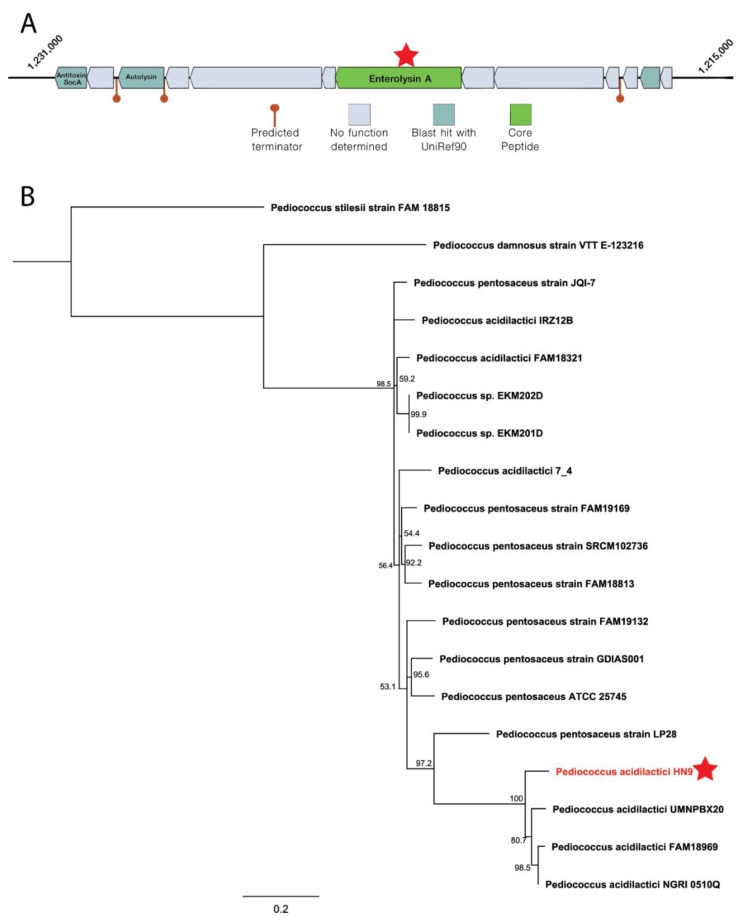
Antimicrobial peptides identified in the HN9 and its phylogenetic relation with other strains. (**A**) Area of Interest (AOI) of *Pediococcus acidilactici* in the genome within a range of 20 kb. Enterolysin A was found as a core protein surrounded by orf00004 (Antitoxin SocA), orf00007 (Autolysin), and orf00023 (uncharacterized protein). The location of Enterolysin A is marked with the red star. (**B**) Phylogenetic analysis of Enterolysin A sequences from the HN9 and other 19 strains. The red star represents the location of *P. acidilactici HN9* in the phylogenetic tree.

To identify the bacteriocin loci, BAGEL4 server was used to perform genome mining and locate the gene encoding the antimicrobial compound. A bacteriocin-encoding locus was identified in the genome of *P. acidilactici* HN9, as shown in Figure 5. Enterolysin A was identified as a core protein in the Area of Interest (AOI) with 100% coverage and 68.7% amino acid sequence identity to the reference sequence (WP_011673272) [72], as shown in Figure S4. It was surrounded by several ORFs, including Antitoxin SocA, Autolysin, and an uncharacterized protein. Enterolysin A is a cell wall-degrading bacteriocin found in several bacterial strains, including *Enterococcus*, *Lactobacillus*, *Streptococcus* [73], and *Pediococcus* [74]. This bacteriocin shows potential for inhibition of enterococci, pediococci, lactococci, and lactobacilli growth. Furthermore, we constructed the phylogenetic tree from Enterolysin A identified in *P. acidilactici* HN9 with other 14 strains as shown in Figure 5B. The lengths and the similarities among these genes varied from 1140 to 3006 bp and 30 to 100%, respectively. The sequence lengths of Enterolysin A from *P. damnosus* and *P. stilesii* are in range of 1–1.8 kbp while the sizes of *P. pentosaceus* and *P. acidilactici* are around 2.9 kbp.

**Figure 6:**
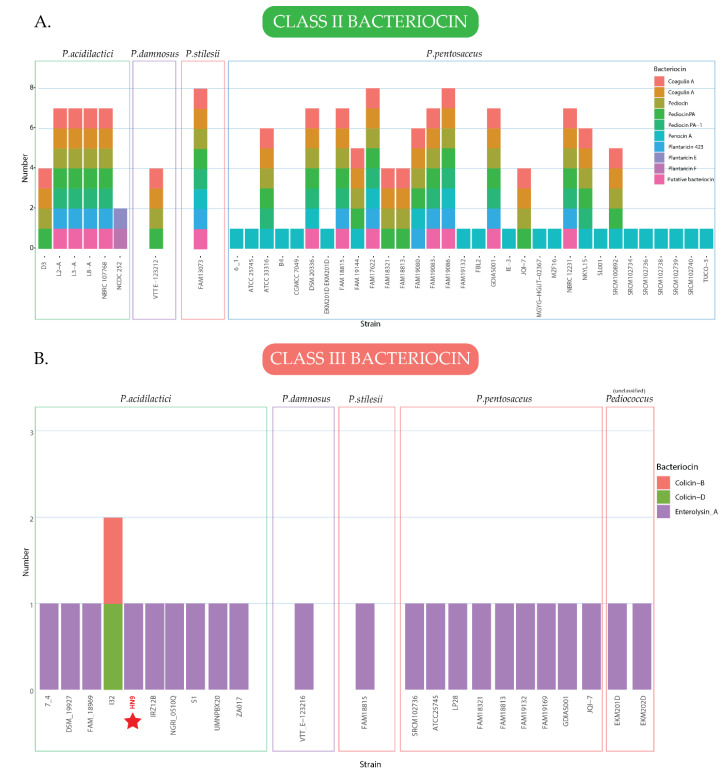
Identification of bacteriocin-encoding genes from all bacterial strains in the genus *Pediococcus* using BLASTX analysis against BAGEL4 database in (**A**) class II and (**B**) class III. The X-axis represents the name of the bacterial strain. The Y-axis represents the total number of bacteriocin identified in each strain. The different colors in each bar represent the different bacteriocins found in each strain, and the rectangle boxes represent the diversity of bacteriocin encoded in different genus. The red star indicates the location of *P. acidilactici HN9*.

In addition, the genomic data of all strains in the genus *Pediococcus* were used to identify bacteriocin genes using BLASTX against the BAGEL4 database. The BLASTX parameters used for the identification included an E-value cutoff-point at 1e-20, and identity percentage and minimum coverage at 70%. Among the 130 strains (including *P. acidilactici* HN9), 40 contained at least one bacteriocin-encoding gene in class II, and 22 strains contained at least one bacteriocin-encoding gene in class III, as shown in Figure 6. *P. Pentosaceus* FAM13073, FAM17622, FAM19086, and GDIAS001 were the most productive bacteriocin producer, encoding 10 different types of bacteriocin. The total number of distinct bacteriocins in class II encoded by the genus *Pediococcus* was 26, with Penocin A, Pediocin, and Pediocin PA being the most common bacteriocins found in this genus, which were produced by 26, 22, and 22 strains, respectively. In contrast, only 4 different class III bacteriocins were identified in the *Pediococcus* group. Enterolysin A was the most common bacteriocin in this genus, which was identified, in 95% of strains encoded class III bacteriocin. In addition, we changed the parameters to scan a wider result by setting the cutoff-point to 1e-10 and identity percentage and minimum coverage to 50%. As a result, we identified 55 strains contained at least one bacteriocin-encoding gene in class II, and 83 contained at least one bacteriocin-encoding gene in class III, as shown in Figure S5.

### 3.8. Identification of Carbohydrate-Active enZyme (CAZyme) in the P. acidilactici HN9 Genome

**Figure 7:**
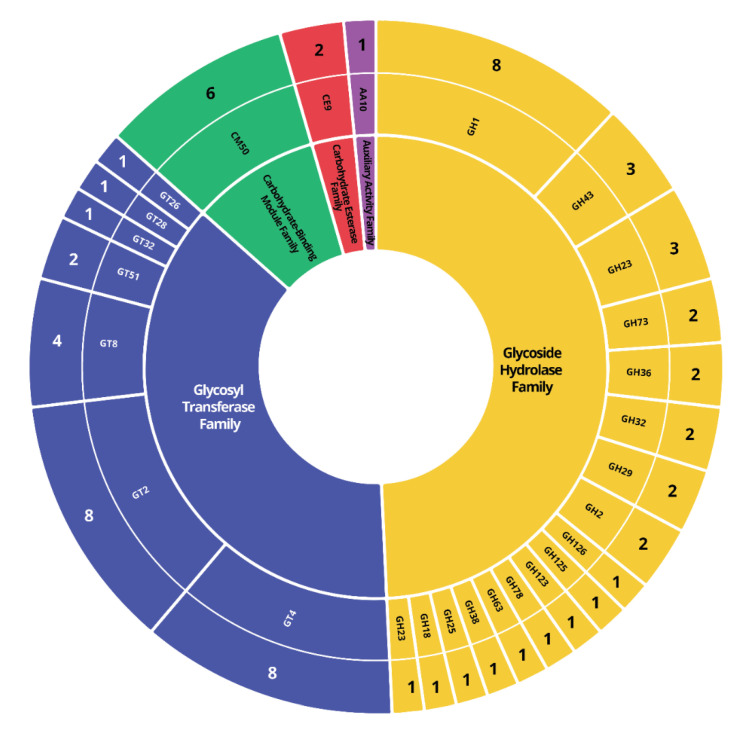
CAZymes distribution in HN9 genome. CAZymes were assigned by searching against the CAZy database using the dbCAN webserver. Different colors represent different classes of CAZymes found in the genome. The representation from the inner to outer rings are, CAZyme classes, CAZyme families, and the number of genes identified in each family, respectively.

The CAZyme is a sequence-based classification of enzymes that enable to synthesize or breakdown complex carbohydrates and glycoconjugates [75]. We identified 67 genes in five families of CAZymes including Glycoside Hydrolase (GH), Glycosyl Transferase (GT), Carbohydrate Esterase (CE), Auxiliary Activity (AA), and Carbohydrate-Binding Module (CBM). The largest family found in the HN9 genome was the GH, which encoded 33 genes in 17 different families (Figure 7). 

### 3.9. In Silico Comparison of Genes Associated with in Meat Fermentation

**Figure 8:**
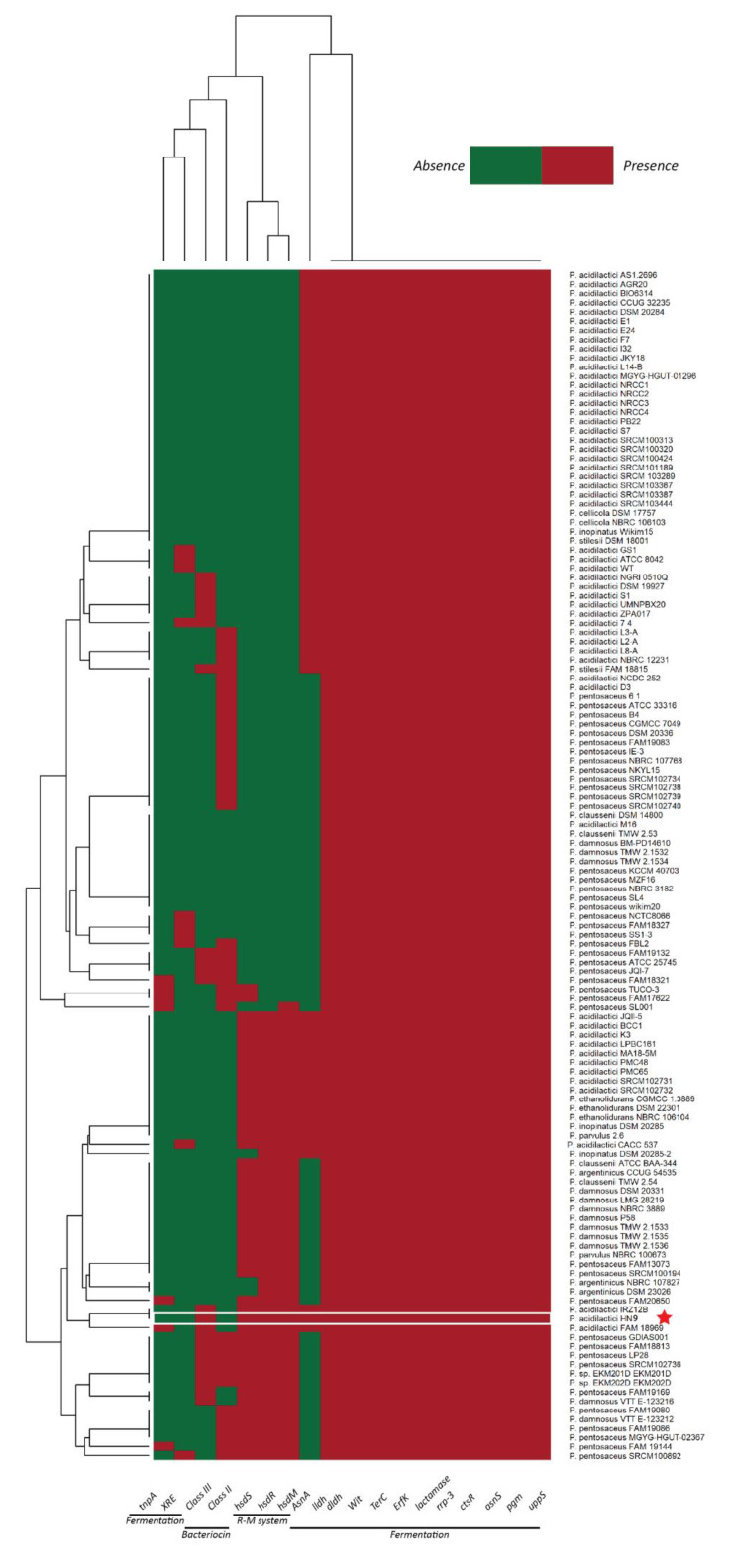
Gene presence-absence heatmap of fermentation related genes. 19 genes were selected to compare the ability in fermentation of Pediococcus 129 strains. Red color represents the presence of the gene and green color indicates the absence of the gene in the genome. The red star indicates the location of *P. acidilactici HN9*.

There was a report that showed some genes highly expressed during meat fermentation including L-Asparaginase 2 (*asnA2*), Phosphoglucomutast (*pgm*), Undecaprenyl pyrophosphate synthetase (*uppS*), Asparaginyl-tRNA synthase (*asnS*), Transposase of IS1520 orfA (*tnpA1*-IS1520), Regulator of class III heat shock genes (*ctsR*), and Response regulator, two-component system (rrp-3) [76]. Therefore, we compared those genes among all bacteria in the *Pediococcus* strains. Also, R-M system-related genes, the presence of the bacteriocin-encoding genes, were included in this analysis to distinguish the ability in the fermentation of each strain, as shown in Figure 8. Not surprisingly, we found that all strains encode the key genes in fermentation, including *ldhL*, *ldhD*. Moreover, every bacterial strain contained various genes such as *pgm*, *uppS*, *asnS*, *ctsR*, *rrp-3,* and several unknown functions of hypothetical proteins. Only *asnA* and tnpA were present in some bacteria. Surprisingly, we found that only 46 strains had *hsdM*, *hsdR*, and *hsdS* which are important genes in the R-M system. We found those three genes in the HN9 genome.

### 3.10. Safety Evaluation and Identification of Antibiotic Resistance Genes

The genomic information of HN9 was used to evaluate the safety of using this strain in food applications. Several tools and databases were used to search for AMR genes, virulence factors, and pathogenic genes. Using the parameters “Perfect hit and Strict hit only” and “High-quality/coverage,” we found no hit in both chromosome and plasmids. Also, the result from the ResFinder showed no hits. However, when the parameter was changed to “Perfect hit, Strict hit and Loose hits”, 122 hits were identified as AMR genes with an identity of matching region range of 21–70 % and coverage of 27–307%. By easing the searching criteria, most of these observed hits were not AMR genes such as *fusA*, *rpoB*, or *gyrB*. Further investigation is warranted to evaluate the antimicrobial susceptibility of this organism. Additionally, the virulence factors were screened using the VFDB database with VFanalyzer [77]. Furthermore, the probability of HN9 being a pathogen was assessed by pathogenicity factor analysis using PathogenFinder. The risk scores of the HN9 genome, plasmid pHN9-1, and pHN9-2 were 0.124, 0.209, and 0.174, respectively; these values are low for being considered as human pathogen compared to known probiotic strains such as *Lactobacillus johnsonii* ZLJ010 (0.193) [78], *Lactobacillus casei* DSM 20011 (0.198) [79], *Lactobacillus reuteri* PNW1 (0.217) [80], and known pathogens such as *Kosakonia radicincitans* DSM 107547 (0.71) [81] and *Enterobacter* sp. C6 (0.755) [82]. Moreover, we found no match of proteins from pathogenic families in HN9. Altogether, the results of our investigations on genomic information of *P. acidilactici* HN9 indicate the safety of HN9 for use in food and beverage applications.

## 4. Discussion

In this study, the complete genome of *P. acidilactici* HN9 and comparative genomic analyses among related species were carried out. The *acidilactici* strains are commonly found in various fermented foods, including vegetables, dairy products, and meat. It has been previously investigated and used as a probiotic to treat constipation [82], diarrhea [5], relieve stress [12], and improve the immune response in human and animals [13]. The functional annotation of HN9 was annotated based on the predicted genes. The subsystems of carbohydrates and protein metabolism were the major subsystem identified in the HN9, which were involved directly in the biological characteristics of this strain. 

Carbohydrate-Active enZymes (CAZymes) are directly involved in biosynthesis, binding, and catabolism of carbohydrates. These enzymes, therefore, play a crucial role in sugar metabolism [75]. The highest number of CAZymes found in the HN9 genome belonged to the GH class. These enzymes are primarily involved in carbohydrate metabolism such as hydrolyzing complex carbohydrates [83,84] and regulating mass degradation during fermentation [85]. In addition, we identified the key enzymes associated with homo-fermentation, including D- and L- lactate dehydrogenases (LDH-D and LDH-L, respectively). These enzymes produce D- and L-lactate, which are key products in the fermentation process of the Embden-Meyerhof-Parnas (EMP) pathway. This suggests that the HN9 can produce important fermentation end-products for producing fermented beef products [86].

The pan-genome analysis was performed to reveal the genomic diversity among *Pediococcus* strains at both genus and species level. Interestingly, we found that the bacterial strains in this genus shared only ~0.7% core genes (226 from 33,568) and contained a very large portion of cloud genes (~45%). Meanwhile, the number of core and cloud genes in only *P. acidilactici* strains were ~6% and ~60%, respectively. The large proportion of cloud genes reveals a remarkably high level of genetic diversity at both genus and species level. Not surprisingly, we found that many bacterial strains contain various mobile genetic elements including plasmids and prophage regions. This can further suggest that HGT could be a key to genomic diversity in this genus [24,74]. Furthermore, the pan-genome analysis also reveals that the trend of pan genes is still increasing and tend to be the “open” genome in both genus and species level as shown in Figure S6. This can indicate that genomes of *Pediococcus* strains are still needed to be sequenced to explore more undiscovered genes and study the genetic diversity among these bacterial strains in this genus.

Bacteriocins are antimicrobial peptides produced by various bacterial species in their ecological niches to inhibit the growth of similar or closely related bacterial strains [87]. Most bacteriocins are produced by gram-positive bacteria, although few are produced by gram-negative bacterial strains [88]. The meta-analysis showed that *Pediococcus* members are potential bacteriocin-producing strains. Most strains in *P. pentosaceus* encode class II and III bacteriocin, while *P. acidilactici* strains mostly encode class III bacteriocin. Class II bacteriocins are small non-lantibiotics peptides comprising approximately 30–70 amino acid residues. Meanwhile, those in class III are large, protein size, bacteriocins [89]. In HN9, we identified a class III bacteriocin, Enterolysin A, which can inhibit the growth of various bacteria including enterococci, pediococci, lactococci, and lactobacilli by degrading the cell wall [73]. Enterolysin A was found in only 20 strains (15.75%) from 129 strains as shown in Figure 6B. In the phylogenetic analysis of strains harboring Enterolysin A, *P. acidilactici* HN9 diverged from other species including *P. pentosaceus*, *P. damnosus,* and *P. stilesii* to form a distinct clade together with three other *P. acidilactici* strains as shown in Figure 5B. In addition, some of *P. acidilactici* strains were found in the same clade as *P. pentosaceus*. This result shows how close these two species are in terms of evolution. However, the diversity of gene content of all bacterial strains is extremely high since different species encode various sizes of the bacteriocin (1–3 kbp). 

Various molecular mechanisms, such as horizontal gene transfer, genome rearrangement, and the presence of mobile genetic elements, could alter the genome stability in bacteria [90,91]. A prophage is a DNA region inserted and integrated into the bacterial DNA chromosome or plasmid [92]. This genetic component is commonly found in various bacterial strains, including *Pediococcus* species [24,74,93]. Interestingly, we found two phage lysins integrated into the prophage region of the genome. Phage lysin has been reported to inhibit pathogenic bacteria [94] and is used in dairy production [95]. The presence of this genetic element could elucidate the mechanism of maintaining stability and adapting to environmental changes.

Bacteriophage infection is a serious problem with fermentation failure. It can easily disrupt commercial fermentation by inhibiting or reducing starter culture activities. Some bacteria have defense mechanisms including Restriction-Modification (R-M) and CRISPRs System to prevent themselves against foreign DNA from other organisms. For *P. acidilactici* HN9, we found two R subunits, two M subunits, and one S subunit of the R-M system. The S subunit is a key genetic element to specify and recognize the DNA sequence. The R subunit cleavages the recognized phage DNA and the M subunit plays an important role in methylation reaction. Interestingly, we found both the R-M system and bacteriocin-encoding gene in the HN9. Among *Pediococcus* strains, we found only 12.4% (16 strains) that have these two properties in the genome. Hence, the HN9 could prevent fermentation failure from the bacteriophage and pathogen infection during the fermentation process. 

In conclusion, our investigation indicates that many *Pediococcus* strains are good candidates for use as a starter culture in the meat/beverage fermentation industry. They encode various genetic elements that are very beneficial and could promote the fermentation process such as the R-M system, CRISPR/Cas system, bacteriocin-encoding genes. These systems play key roles in preventing the integration of virulence factors, pathogenic genes, antimicrobial resistance (AMR) genes via horizontal gene transfer during its evolution. Also, they can inhibit and prevent the growth of spoilage bacteria and foodborne pathogens. For the HN9 genome, we found no match of proteins from pathogenic families and antibiotic resistance genes and contain many beneficial genes that help promote the meat fermentation for producing functional fermented meats. These results of our investigation suggest that *P. acidilactici* HN9 could be a great potential starter culture for food and beverage fermentation applications.

## Figures and Tables

**Table 1:** Genomic features of *P. acidilactici* HN9.

	Chromosome	Plasmids	
		pHN9-1	pHN9-2
Size (bp)	2,034,522	42,239	30,711
DNA G+C content (%)	42.1	42.6	36.0
Number of CDS	2013	54	42
rRNA	15	0	0
tRNA	56	0	0
RAST subsystems	200	2	2
CRISPR	2	0	0
Prophages	3	0	2
Integrative and conjugative elements (ICEs)	0	1	0
Antimicrobial resistance (AMR) gene	0	0	0
Probability of being a human pathogen	0.124	0.209	0.174

**Table 2:** Prophage information of *Pediococcus acidilactici* HN9.

Region	Region Length	Completeness	TotalCDS	Region Position	Most Common Phage	GC %
1	16.6Kb	incomplete	30	665577- 682211	PHAGE_Lactob_Lc_Nu_NC_007501(3)	37.16
2	21.9Kb	intact	24	681401-703303	PHAGE_Strept_Abc2_NC_013645(6)	39.82
3	43.5Kb	intact	56	1210062-1253564	PHAGE_Lactob_iLp1308_NC_028911(10)	39.46

## Data Availability

*Pediococcus acidilactici* HN9 was deposited in BioProject and BioSample PRJNA663983, SAMN16191522. The assemblies of the genome, plasmid pHN9-1, and pHN9-2 were deposited in GenBank; CP061715-CP061717. The raw sequence reads of PacBio and Illumina were deposited in the Sequence Read Archive (SRA) database, SRR12768941-SRR12768942.

## Supplementary Materials

The following are available online at https://www.mdpi.com/2076-2607/9/1/50/s1, Figure S1: Subsystem information of the HN9 based on the RAST annotation server, Figure S2: Phylogenetic analysis of *Pediococcus acidilactici* HN9 and other strains at the genus level, based on 226 core genes from 129 strains. The tree was constructed using the neighbor-joining method with a bootstrap of 500 replications by concatenating the single-copy marker genes computed using Roary. The scale bar represents an evolutionary distance, Figure S3: Phylogenetic analysis of *Pediococcus acidilactici* HN9 and other strains at the species level, Figure S4: Sequence alignment of Enterolysin A from *Pediococcus pentosaceus* ATCC 25745 with Enterolysin A identified in the HN9, Figure S5: Identification of bacteriocin-encoding genes from all bacterial strains in the genus *Pediococcus*, Figure S6: Pan-genome and core-genome of *Pediococcus* 129 strains. The pan-genome and core-genome plot are represented by the accumulated frequency of new genes and the core-genomes against the number of genomes added, respectively, Table S1: The information of strain used in this study, Table S2: Metadata of all strains used in this study.
