# Supplementary material for: Genomic Insight into Pediococcus acidilactici HN9, a Potential Probiotic Strain Isolated from the Traditional Thai-Style Fermented Beef Nhang
Source: Microorganisms. 2020 Dec 27;9(1):50. doi: 10.3390/microorganisms9010050 (PMC7823806; doi:10.3390/microorganisms9010050)
Supplement: Supplementary file 1 [file microorganisms-09-00050-s001.zip › Supplementary/supplementary table.pdf]

Table S1 The information of strain used in this study.

Strains	Assembly Accession	Status	Size (Mb)	Assembly Coverage	Submitter Organization
**HN9*** (This study)	**CP061715-CP061717**	**Complete**	**2.14**	**500**	**Prince of Songkla University**
ZPA017	GCF_001767275.1	Complete	2.13	N/A	Beijing Academy of Agriculture and Forestry Sciences
BCC1	GCF_001922325.1	Complete	2.17	415	China Agricultural University
SRCM100424	GCF_002173575.1	Complete	2.09	628	Microbial Institute for Fermentation Industry
SRCM100313	GCF_002173595.1	Complete	2.11	607	Microbial Institute for Fermentation Industry
SRCM101189	GCF_002174215.1	Complete	2.09	1338	Microbial Institute for Fermentation Industry
PB22	GCF_003957355.1	Complete	1.95	416	Korea Food Research Institute
SRCM103387	GCF_004101585.1	Complete	2.03	310.93	Microbial Institute for Fermentation Industry
SRCM103367	GCF_004102605.1	Complete	1.96	234.7	Microbial Institute for Fermentation Industry
SRCM103444	GCF_004103635.1	Complete	19.70	346.26	Microbial Institute for Fermentation Industry
ATCC 8042	GCF_004355265.1	Complete	2.01	531	Seoul National University
JQII-5	GCF_006770685.1	Complete	2.08	200	BGI
SRCM102731	GCF_009913875.1	Complete	2.06	355	Microbial Institute for Fermentation Industry
SRCM102732	GCF_009913895.1	Complete	2.07	447	Microbial Institute for Fermentation Industry
CACC 537	GCF_010092385.1	Complete	2.03	12	Center for Industrialization of Agricultural and Livestock Microorganisms
PMC48	GCF_011604585.1	Complete	2.04	209.81	Probiotics Microbiome Convergence Center
PMC65	GCF_013127755.1	Complete	2.04	482.33	Probiotics Microbiome Convergence Center
DSM 20284	GCF_000146325.1	Scaffold	1.94	130	Baylor College of Medicine
7_4	GCF_000163095.1	Scaffold	2.01	N/A	Broad Institute
D3	GCF_000380265.1	Scaffold	1.96	567	Texas A&M University
DSM 19927	GCF_001437115.1	Scaffold	2.04	100	Shanghai Majorbio
LPBC161	GCF_004022295.1	Scaffold	1.97	11	UFPR
L8-A	GCF_004099675.1	Scaffold	1.94	142	Texas Tech University
L3-A	GCF_004099705.1	Scaffold	1.94	140	Texas Tech University
L2-A	GCF_004099815.1	Scaffold	1.94	214	Texas Tech University
L14-B	GCF_004100005.1	Scaffold	1.93	67	Texas Tech University
IRZ12B	GCF_013375165.1	Scaffold	2.05	258	Padova university
MGYG-HGUT-01296	GCF_902373655.1	Scaffold	1.94	10	EMG
SRCM100320	GCF_001672605.1	Contig	2.07	758	Microbial Institute for Fermentation Industry
NRCC4	GCF_001867265.1	Contig	1.45	12	ICAR-National Research Centre on Camel
JKY18	GCF_001868705.1	Contig	1.87	788	Anhui University
UMNPBX20	GCF_002553675.1	Contig	2.03	50	University of Minnesota
GS1	GCF_003571945.1	Contig	2.00	120	Kangwon National University
WT	GCF_003591195.1	Contig	2.02	160	Kangwon National University
SRCM103289	GCF_004054355.1	Contig	2.09	393.98	Chunlab
FAM 18969	GCF_005864375.1	Contig	2.07	60	Agroscope
NBRC 12231	GCF_006539025.1	Contig	1.93	298	National Institute of Technology and Evaluation Biological Resource Center
E1	GCF_009910835.1	Contig	2.01	20	University of Surrey
M16	GCF_011009585.1	Contig	1.82	5	University of Surrey
I32	GCF_011009675.1	Contig	2.03	15	University of Surrey
E24	GCF_011009695.1	Contig	2.01	15	University of Surrey
F7	GCF_011009715.1	Contig	2.09	15	University of Surrey
CCUG 32235	GCF_012396515.1	Contig	1.93	162.8	Bacterial Special Pathogens Branch (CDC)
BIO6314	GCF_013249135.1	Contig	1.92	876	Bioprox
S7	GCF_900163535.1	Contig	2.01	448	MICALIS

Table S2 Metadata of all strains used in this study.

AssemblyAccession	AssemblyName	Taxid	Organism	SpeciesTaxid	SpeciesName	AssemblyStatus	BioprojectAccn	BioprojectId	BioprojectAccn2	BioprojectId3	BioSampleAccn	BioSampleId	Sub_type	Sub_value	Coverage	SubmitterOrganization	RefSeq_category	string4	Genbank	RefSeq	Similarity	ContigN50	ScaffoldN50
GCF_002982135.1	ASM298213v1	114090	Pediococcus inopinatus (firmicutes)	114090	Pediococcus inopinatus	Complete Genome	PRJNA376812	376812	PRJNA224116	224116	SAMN06447728	6447728	strain	DSM 20285	396.94	Kyungpook National University	representative genome	from-type	GCA_002982135.1	GCF_002982135.1	identical	2137091	2137091
GCF_001611155.1	ASM161115v1	51663	Pediococcus damnosus (firmicutes)	51663	Pediococcus damnosus	Complete Genome	PRJNA290141	290141	PRJNA224116	224116	SAMN03876485	3876485	strain	TMW 2.1536	137	Technische Universitaet Muenchen	representative genome	full-genome-representation	GCA_001611155.1	GCF_001611155.1	identical	2125430	2125430
GCF_013127755.1	ASM1312775v1	1254	Pediococcus acidilactici (firmicutes)	1254	Pediococcus acidilactici	Complete Genome	PRJNA632644	632644	PRJNA224116	224116	SAMN14916503	14916503	strain	PMC 65	482.33	Probiotics Microbiome Convergence Center	representative genome	full-genome-representation	GCA_013127755.1	GCF_013127755.1	identical	2044083	2044083
GCF_001702215.1	ASM170221v1	187452	Pediococcus claussenii (firmicutes)	187452	Pediococcus claussenii	Complete Genome	PRJNA290141	290141	PRJNA224116	224116	SAMN04505731	4505731	strain	TMW 2.53	192	Technische Universitaet Muenchen	representative genome	full-genome-representation	GCA_001702215.1	GCF_001702215.1	identical	1889038	1889038
GCF_000014505.1	ASM1450v1	278197	Pediococcus pentosaceus ATCC 25745 (firmicutes)	1255	Pediococcus pentosaceus	Complete Genome	PRJNA398	398	PRJNA224116	224116	SAMN02598525	2598525	strain	ATCC 25745	0	US DOE Joint Genome Institute (JGI), The Lactic Acid Bacteria Genome Consortium and Fidelity Systems Inc.	representative genome	full-genome-representation	GCA_000014505.1	GCF_000014505.1	identical	1832387	1832387
GCF_001438655.1	ASM143865v1	319652	Pediococcus cellicola (firmicutes)	319652	Pediococcus cellicola	Contig	PRJNA222257	222257	PRJNA224116	224116	SAMN02797811	2797811	strain	DSM 17757	100	Shanghai Majorbio	representative genome	from-type	GCA_001438655.1	GCF_001438655.1	identical	218167	218167
GCF_001437075.1	ASM143707v1	331679	Pediococcus stilesii (firmicutes)	331679	Pediococcus stilesii	Contig	PRJNA222257	222257	PRJNA224116	224116	SAMN02797812	2797812	strain	DSM 18001	100	Shanghai Majorbio	representative genome	from-type	GCA_001437075.1	GCF_001437075.1	identical	98964	98964

GCF_001437405.1	ASM143740v1	319653	Pediococcus ethanolidurans (firmicutes)	319653	Pediococcus ethanolidurans	Contig	PRJNA222257	222257	PRJNA224116	224116	SAMN02797818	2797818	strain	DSM 22301	100	Shanghai Majorbio	representative genome	from-type	GCA_001437405.1	GCF_001437405.1	identical	94848	94848
GCF_012396535.1	ASM1239653v1	480391	Pediococcus argentinicus (firmicutes)	480391	Pediococcus argentinicus	Contig	PRJNA622446	622446	PRJNA224116	224116	SAMN14517845	14517845	strain	CCUG 54535	199	Bacterial Special Pathogens Branch (CDC)	representative genome	from-type	GCA_012396535.1	GCF_012396535.1	identical	47517	47517
GCF_007990205.1	ASM799020v1	54062	Pediococcus parvulus (firmicutes)	54062	Pediococcus parvulus	Contig	PRJDB6092	396402	PRJNA224116	224116	SAMD00166260	11337974	strain	NBRC 100673	124	National Institute of Technology and Evaluation	representative genome	from-type	GCA_007990205.1	GCF_007990205.1	identical	40210	40210
GCF_000237995.1	ASM23799v2	701521	Pediococcus claussenii ATCC BAA-344 (firmicutes)	187452	Pediococcus claussenii	Complete Genome	PRJNA42741	42741	PRJNA224116	224116	SAMN02604240	2604240	strain	ATCC BAA-344	0	University of Saskatchewan	na	from-type	GCA_000237995.2	GCF_000237995.1	identical	1829111	1829111
GCF_001611135.1	ASM161113v1	51663	Pediococcus damnosus (firmicutes)	51663	Pediococcus damnosus	Complete Genome	PRJNA290141	290141	PRJNA224116	224116	SAMN03876484	3876484	strain	TMW 2.1535	142	Technische Universitaet Muenchen	na	full-genome-representation	GCA_001611135.1	GCF_001611135.1	identical	2247318	2247318
GCF_001611115.1	ASM161111v1	51663	Pediococcus damnosus (firmicutes)	51663	Pediococcus damnosus	Complete Genome	PRJNA290141	290141	PRJNA224116	224116	SAMN03876483	3876483	strain	TMW 2.1534	138	Technische Universitaet Muenchen	na	full-genome-representation	GCA_001611115.1	GCF_001611115.1	identical	2172287	2172287
GCF_001611075.1	ASM161107v1	51663	Pediococcus damnosus (firmicutes)	51663	Pediococcus damnosus	Complete Genome	PRJNA290141	290141	PRJNA224116	224116	SAMN03876482	3876482	strain	TMW 2.1533	92	Technische Universitaet Muenchen	na	full-genome-representation	GCA_001611075.1	GCF_001611075.1	identical	2149374	2149374
GCF_001767275.1	ASM176727v1	1254	Pediococcus acidilactici (firmicutes)	1254	Pediococcus acidilactici	Complete Genome	PRJNA312971	312971	PRJNA224116	224116	SAMN04515708	4515708	strain	ZPA017	0	Beijing Academy of Agriculture and Forestry Sciences	na	full-genome-representation	GCA_001767275.1	GCF_001767275.1	identical	2131361	2131361

GCF_001922325.1	ASM192232v1	1254	Pediococcus acidilactici (firmicutes)	1254	Pediococcus acidilactici	Complete Genome	PRJNA357663	357663	PRJNA224116	224116	SAMN06052263	6052263	strain	BCC1	415	China Agricultural University	na	full-genome-representation	GCA_001922325.1	GCF_001922325.1	identical	2096059	2096059
GCF_006770685.1	ASM677068v1	1254	Pediococcus acidilactici (firmicutes)	1254	Pediococcus acidilactici	Complete Genome	PRJNA412124	412124	PRJNA224116	224116	SAMN07701376	7701376	strain	JQII-5	200	BGI	na	full-genome-representation	GCA_006770685.1	GCF_006770685.1	identical	2085679	2085679
GCF_001611035.1	ASM161103v1	51663	Pediococcus damnosus (firmicutes)	51663	Pediococcus damnosus	Complete Genome	PRJNA290141	290141	PRJNA224116	224116	SAMN03876481	3876481	strain	TMW 2.1532	144	Technische Universitaet Muenchen	na	full-genome-representation	GCA_001611035.1	GCF_001611035.1	identical	2073735	2073735
GCF_011604585.1	ASM1160458v1	1254	Pediococcus acidilactici (firmicutes)	1254	Pediococcus acidilactici	Complete Genome	PRJNA612145	612145	PRJNA224116	224116	SAMN14362951	14362951	strain	PMC48	209.81	Probiotics Microbiome Convergence Center	na	full-genome-representation	GCA_011604585.1	GCF_011604585.1	identical	2043929	2043929
GCF_010092385.1	ASM1009238v1	1254	Pediococcus acidilactici (firmicutes)	1254	Pediococcus acidilactici	Complete Genome	PRJNA601629	601629	PRJNA224116	224116	SAMN13871425	13871425	strain	CACC 537	12	Center for Industrialization of Agricultural and Livestock Microorganisms	na	full-genome-representation	GCA_010092385.1	GCF_010092385.1	identical	2035984	2035984
GCF_002174215.1	ASM217421v1	1254	Pediococcus acidilactici (firmicutes)	1254	Pediococcus acidilactici	Complete Genome	PRJNA388027	388027	PRJNA224116	224116	SAMN07167785	7167785	strain	SRCM101189	1338	Microbial Institute for Fermentation Industry	na	full-genome-representation	GCA_002174215.1	GCF_002174215.1	identical	2025732	2025732
GCF_002173575.1	ASM217357v1	1254	Pediococcus acidilactici (firmicutes)	1254	Pediococcus acidilactici	Complete Genome	PRJNA386762	386762	PRJNA224116	224116	SAMN07126147	7126147	strain	SRCM100424	628	Microbial Institiue for Fermentation Industry	na	full-genome-representation	GCA_002173575.1	GCF_002173575.1	identical	2025714	2025714
GCF_002173595.1	ASM217359v1	1254	Pediococcus acidilactici (firmicutes)	1254	Pediococcus acidilactici	Complete Genome	PRJNA386761	386761	PRJNA224116	224116	SAMN07125954	7125954	strain	SRCM100313	607	Microbial Institiue for Fermentation Industry	na	full-genome-representation	GCA_002173595.1	GCF_002173595.1	identical	2025575	2025575
GCF_009913895.1	ASM991389v1	1254	Pediococcus acidilactici	1254	Pediococcus acidilactici	Complete Genome	PRJNA438180	438180	PRJNA224116	224116	SAMN08707607	8707607	strain	SRCM102732	447	Microbial Institute for Fermentati	na	full-genome-repres	GCA_009913895.1	GCF_009913895.1	identical	2018117	2018117

			(firmicutes)													on Industry		entation					
GCF_009913875.1	ASM991387v1	1254	Pediococcus acidilactici (firmicutes)	1254	Pediococcus acidilactici	Complete Genome	PRJNA438180	438180	PRJNA224116	224116	SAMN08707606	8707606	strain	SRCM102731	355	Microbial Institute for Fermentation Industry	na	full-genome-representation	GCA_009913875.1	GCF_009913875.1	identical	2015015	2015015
GCF_004355265.1	ASM435526v1	1254	Pediococcus acidilactici (firmicutes)	1254	Pediococcus acidilactici	Complete Genome	PRJNA503305	503305	PRJNA224116	224116	SAMN10358339	10358339	strain	ATCC 8042	531	Seoul National University	na	full-genome-representation	GCA_004355265.1	GCF_004355265.1	identical	2009598	2009598
GCF_004101585.1	ASM410158v1	1254	Pediococcus acidilactici (firmicutes)	1254	Pediococcus acidilactici	Complete Genome	PRJNA515148	515148	PRJNA224116	224116	SAMN10743318	10743318	strain	SRCM103387	310.93	Microbial Institute for Fermentation Industry	na	full-genome-representation	GCA_004101585.1	GCF_004101585.1	identical	2001079	2001079
GCF_004103635.1	ASM410363v1	1254	Pediococcus acidilactici (firmicutes)	1254	Pediococcus acidilactici	Complete Genome	PRJNA515377	515377	PRJNA224116	224116	SAMN10754287	10754287	strain	SRCM103444	346.26	Microbial Institute for Fermentation Industry	na	full-genome-representation	GCA_004103635.1	GCF_004103635.1	identical	1970727	1970727
GCF_003957355.1	ASM395735v1	1254	Pediococcus acidilactici (firmicutes)	1254	Pediococcus acidilactici	Complete Genome	PRJNA422477	422477	PRJNA224116	224116	SAMN08180273	8180273	strain	PB22	416	Korea Food Research Institute	na	full-genome-representation	GCA_003957355.1	GCF_003957355.1	identical	1955616	1955616
GCF_001702235.1	ASM170223v1	187452	Pediococcus claussenii (firmicutes)	187452	Pediococcus claussenii	Complete Genome	PRJNA290141	290141	PRJNA224116	224116	SAMN04505732	4505732	strain	TMW 2.54	146	Technische Universitaet Muenchen	na	full-genome-representation	GCA_001702235.1	GCF_001702235.1	identical	1886832	1886832
GCF_004102605.1	ASM410260v1	1254	Pediococcus acidilactici (firmicutes)	1254	Pediococcus acidilactici	Complete Genome	PRJNA515147	515147	PRJNA224116	224116	SAMN10743317	10743317	strain	SRCM103367	234.7	Microbial Institute for Fermentation Industry	na	full-genome-representation	GCA_004102605.1	GCF_004102605.1	identical	1852892	1852892
GCF_007923185.1	ASM792318v1	1255	Pediococcus pentosaceus (firmicutes)	1255	Pediococcus pentosaceus	Complete Genome	PRJNA526333	526333	PRJNA224116	224116	SAMN11094516	11094516	strain	SL001	5758	Hunan Normal University	na	full-genome-representation	GCA_007923185.1	GCF_007923185.1	identical	1842476	1842476
GCF_009791435.1	ASM979143v1	1255	Pediococcus pentos	1255	Pediococcus	Complete	PRJNA596038	596038	PRJNA224116	224116	SAMN13611447	13611447	strain	GDIA S001	250	Guangdong Academy	na	full-genome-	GCA_009791435.1	GCF_009791435.1	identical	1831351	1831351

			aceus (firmicutes)		pentosaceus	Genome										of Agricultural Sciences		representation					
GCF_009914035.1	ASM991403v1	1255	Pediococcus pentosaceus (firmicutes)	1255	Pediococcus pentosaceus	Complete Genome	PRJNA438180	438180	PRJNA224116	224116	SAMN08707615	8707615	strain	SRCM102740	789	Microbial Institute for Fermentation Industry	na	full-genome-representation	GCA_009914035.1	GCF_009914035.1	identical	1818150	1818150
GCF_009913995.1	ASM991399v1	1255	Pediococcus pentosaceus (firmicutes)	1255	Pediococcus pentosaceus	Complete Genome	PRJNA438180	438180	PRJNA224116	224116	SAMN08707613	8707613	strain	SRCM102738	750	Microbial Institute for Fermentation Industry	na	full-genome-representation	GCA_009913995.1	GCF_009913995.1	identical	1818149	1818149
GCF_009914015.1	ASM991401v1	1255	Pediococcus pentosaceus (firmicutes)	1255	Pediococcus pentosaceus	Complete Genome	PRJNA438180	438180	PRJNA224116	224116	SAMN08707614	8707614	strain	SRCM102739	362	Microbial Institute for Fermentation Industry	na	full-genome-representation	GCA_009914015.1	GCF_009914015.1	identical	1818149	1818149
GCF_000496265.1	ASM49626v1	1408206	Pediococcus pentosaceus SL4 (firmicutes)	1255	Pediococcus pentosaceus	Complete Genome	PRJNA222573	222573	PRJNA224116	224116	SAMN02603952	2603952	strain	SL4	3700	The Technical University of Denmark	na	full-genome-representation	GCA_000496265.1	GCF_000496265.1	identical	1789138	1789138
GCF_002173535.1	ASM217353v1	1255	Pediococcus pentosaceus (firmicutes)	1255	Pediococcus pentosaceus	Complete Genome	PRJNA386763	386763	PRJNA224116	224116	SAMN07126152	7126152	strain	SRCM100892	594	Microbial Institiue for Fermentation Industry	na	full-genome-representation	GCA_002173535.1	GCF_002173535.1	identical	1785266	1785266
GCF_002982155.1	ASM298215v1	1255	Pediococcus pentosaceus (firmicutes)	1255	Pediococcus pentosaceus	Complete Genome	PRJNA376813	376813	PRJNA224116	224116	SAMN06447729	6447729	strain	KCCM 40703	460.75	Kyungpook National University	na	full-genome-representation	GCA_002982155.1	GCF_002982155.1	identical	1758362	1758362
GCF_002202155.1	ASM220215v1	1255	Pediococcus pentosaceus (firmicutes)	1255	Pediococcus pentosaceus	Complete Genome	PRJNA390207	390207	PRJNA224116	224116	SAMN07224387	7224387	strain	SRCM100194	2643	Microbial Institute for Fermentation Industry	na	full-genome-representation	GCA_002202155.1	GCF_002202155.1	identical	1757573	1757573
GCF_009913955.1	ASM991395v1	1255	Pediococcus pentosaceus (firmicutes)	1255	Pediococcus pentosaceus	Complete Genome	PRJNA438180	438180	PRJNA224116	224116	SAMN08707611	8707611	strain	SRCM102736	568	Microbial Institute for Fermentation Industry	na	full-genome-representation	GCA_009913955.1	GCF_009913955.1	identical	1756764	1756764

GCF_003429405.1	ASM342940v1	1255	Pediococcus pentosaceus (firmicutes)	1255	Pediococcus pentosaceus	Complete Genome	PRJNA399825	399825	PRJNA224116	224116	SAMN07551496	7551496	strain	SS1-3	341	Korea Food Research Institute	na	full-genome-representation	GCA_003429405.1	GCF_003429405.1	identical	1752312	1752312
GCF_001411765.2	ASM141176v2	1255	Pediococcus pentosaceus (firmicutes)	1255	Pediococcus pentosaceus	Complete Genome	PRJNA260124	260124	PRJNA224116	224116	SAMN04017317	4017317	strain	wikim20	621.6	WIKIM	na	full-genome-representation	GCA_001411765.2	GCF_001411765.2	identical	1739283	1739283
GCF_006770865.1	ASM677086v1	1255	Pediococcus pentosaceus (firmicutes)	1255	Pediococcus pentosaceus	Complete Genome	PRJNA412135	412135	PRJNA224116	224116	SAMN07701459	7701459	strain	JQI-7	200	BGI	na	full-genome-representation	GCA_006770865.1	GCF_006770865.1	identical	1732880	1732880
GCF_009930955.1	ASM993095v1	1255	Pediococcus pentosaceus (firmicutes)	1255	Pediococcus pentosaceus	Complete Genome	PRJNA438180	438180	PRJNA224116	224116	SAMN08707609	8707609	strain	SRCM102734	487	Microbial Institute for Fermentation Industry	na	full-genome-representation	GCA_009930955.1	GCF_009930955.1	identical	1707614	1707614
GCF_000146325.1	ASM14632v1	862514	Pediococcus acidilactici DSM 20284 (firmicutes)	1254	Pediococcus acidilactici	Scaffold	PRJNA50527	50527	PRJNA224116	224116	SAMN00116807	116807	strain	DSM 20284	130	Baylor College of Medicine	na	from-type	GCA_000146325.1	GCF_000146325.1	identical	425094	1937622
GCF_012396515.1	ASM1239651v1	1254	Pediococcus acidilactici (firmicutes)	1254	Pediococcus acidilactici	Contig	PRJNA622446	622446	PRJNA224116	224116	SAMN14517844	14517844	strain	CCUG 32235	162.8	Bacterial Special Pathogens Branch (CDC)	na	from-type	GCA_012396515.1	GCF_012396515.1	identical	446249	446249
GCF_001437115.1	ASM143711v1	1254	Pediococcus acidilactici (firmicutes)	1254	Pediococcus acidilactici	Scaffold	PRJNA222257	222257	PRJNA224116	224116	SAMN02797813	2797813	strain	DSM 19927	100	Shanghai Majorbio	na	from-type	GCA_001437115.1	GCF_001437115.1	identical	318866	318866
GCF_004354495.1	ASM435449v1	1255	Pediococcus pentosaceus (firmicutes)	1255	Pediococcus pentosaceus	Contig	PRJNA434256	434256	PRJNA224116	224116	SAMN08543181	8543181	strain	ATCC 33316	60	Carlsberg Research Laboratory	na	from-type	GCA_004354495.1	GCF_004354495.1	identical	302347	302347
GCF_001437285.1	ASM143728v1	1255	Pediococcus pentosaceus	1255	Pediococcus pentosaceus	Contig	PRJNA222257	222257	PRJNA224116	224116	SAMN02797817	2797817	strain	DSM 20336	100	Shanghai Majorbio	na	from-type	GCA_001437285.1	GCF_001437285.1	identical	295919	295919

			(firmic utes)																				
GCF_001 436905.1	ASM14 3690v1	187 452	Pedioc occus clauss enii (firmic utes)	18745 2	Pedioc occus clauss enii	Scaffol d	PRJNA 222257	22225 7	PRJNA 224116	22411 6	SAMN0 2797810	27978 10	strai n	DSM 14800	100	Shanghai Majorbio	na	from-type	GCA_0 0143690 5.1	GCF_00 1436905 .1	iden tical	2042 00	21983 8
GCF_000 319265.1	Plo_1.0	122 928 1	Pedioc occus acidila ctici NGRI 0510Q (firmic utes)	1254	Pedioc occus acidila ctici	Contig	PRJDB 499	17440 8	PRJNA 224116	22411 6	SAMD0 0036646	40916 11	strai n	NGRI 0510 Q	232	Microbial Genetics, Institute of Genetic Resources, Faculty of Agricultur e, Kyushu University	na	from-type	GCA_0 0031926 5.1	GCF_00 0319265 .1	iden tical	1023 22	10232 2
GCF_900 111205.1	IMG-taxon 261727 0883 annotat ed assemb ly	319 653	Pedioc occus ethano lidura ns (firmic utes)	31965 3	Pedioc occus ethano lidura ns	Scaffol d	PRJEB 17023	35120 7	PRJNA 224116	22411 6	SAMN0 4487973	44879 73	strai n	CGM CC 1.388 9	624	DOE - JOINT GENOME INSTITUT E	na	from-type	GCA_9 0011120 5.1	GCF_90 0111205 .1	iden tical	7443 3	10084 4
GCF_001 437605.1	ASM14 3760v1	480 391	Pedioc occus argent inicus (firmic utes)	48039 1	Pedioc occus argent inicus	Scaffol d	PRJNA 222257	22225 7	PRJNA 224116	22411 6	SAMN0 2797819	27978 19	strai n	DSM 23026	100	Shanghai Majorbio	na	from-type	GCA_0 0143760 5.1	GCF_00 1437605 .1	iden tical	4365 7	43657
GCF_001 438725.1	ASM14 3872v1	114 090	Pedioc occus inopin atus (firmic utes)	11409 0	Pedioc occus inopin atus	Contig	PRJNA 222257	22225 7	PRJNA 224116	22411 6	SAMN0 2797814	27978 14	strai n	DSM 20285	100	Shanghai Majorbio	na	from-type	GCA_0 0143872 5.1	GCF_00 1438725 .1	iden tical	3792 3	37923
GCF_001 437255.1	ASM14 3725v1	516 63	Pedioc occus damn osus (firmic utes)	51663	Pedioc occus damn osus	Contig	PRJNA 222257	22225 7	PRJNA 224116	22411 6	SAMN0 2797815	27978 15	strai n	DSM 20331	100	Shanghai Majorbio	na	from-type	GCA_0 0143725 5.1	GCF_00 1437255 .1	iden tical	2085 9	20859
GCF_004 054355.1	ASM40 5435v1	125 4	Pedioc occus acidila ctici (firmic utes)	1254	Pedioc occus acidila ctici	Contig	PRJNA 514932	51493 2	PRJNA 224116	22411 6	SAMN1 0737798	10737 798	strai n	SRC M 10328 9	393. 98	Chunlab	na	full-geno me-repres entati on	GCA_0 0405435 5.1	GCF_00 4054355 .1	iden tical	2022 494	20224 94
GCF_009 809595.1	ASM98 0959v1	125 5	Pedioc occus pentos aceus	1255	Pedioc occus pentos aceus	Contig	PRJNA 576774	57677 4	PRJNA 224116	22411 6	SAMN1 3008348	13008 348	strai n	FAM 19132	124	Agroscope	na	full-geno me-repres	GCA_0 0980959 5.1	GCF_00 9809595 .1	iden tical	1906 512	19065 12

			(firmicutes)															entation					
GCF_000526815.1	ASM52681v1	1384067	Pediococcus acidilactici AGR20 (firmicutes)	1254	Pediococcus acidilactici	Contig	PRJNA214531	214531	PRJNA224116	224116	SAMN02584994	2584994	strain	AGR20	0	DOE Joint Genome Institute	na	full-genome-representation	GCA_000526815.1	GCF_000526815.1	identical	1850049	1850049
GCF_900454755.1	54332_C01	1255	Pediococcus pentosaceus (firmicutes)	1255	Pediococcus pentosaceus	Contig	PRJEB6403	251923	PRJNA224116	224116	SAMEA101173918	7674453	strain	NCTC8066	100	SC	na	full-genome-representation	GCA_900454755.1	GCF_900454755.1	identical	1785489	1785489
GCF_000708635.1	PenPed1.0	1460385	Pediococcus pentosaceus CGMCC 7049 (firmicutes)	1255	Pediococcus pentosaceus	Contig	PRJNA237570	237570	PRJNA224116	224116	SAMN02639794	2639794	strain	CGMCC 7049	50	State Key Laboratory for Diagnosis and Treatment of Infectious Disease, The First Affiliated Hospital, Zhejiang University	na	full-genome-representation	GCA_000708635.1	GCF_000708635.1	identical	476563	476563
GCF_902386405.1	UHGG_MGYG-HGUT-02367	1255	Pediococcus pentosaceus (firmicutes)	1255	Pediococcus pentosaceus	Contig	PRJEB33885	559126	PRJNA224116	224116	SAMEA5851871	12520869			10	EMG	na	full-genome-representation	GCA_902386405.1	GCF_902386405.1	identical	476563	476563
GCF_009808655.1	ASM980865v1	1255	Pediococcus pentosaceus (firmicutes)	1255	Pediococcus pentosaceus	Contig	PRJNA576774	576774	PRJNA224116	224116	SAMN13008394	13008394	strain	FAM18327	18	Agroscope	na	full-genome-representation	GCA_009808655.1	GCF_009808655.1	identical	448591	448591
GCF_004099705.1	ASM409970v1	1254	Pediococcus acidilactici (firmicutes)	1254	Pediococcus acidilactici	Scaffold	PRJNA515210	515210	PRJNA224116	224116	SAMN10744158	10744158	strain	L3-A	140	Texas Tech University	na	full-genome-representation	GCA_004099705.1	GCF_004099705.1	identical	434882	434882
GCF_902373655.1	MGYG-HGUT-01296	1254	Pediococcus acidilactici	1254	Pediococcus acidilactici	Scaffold	PRJEB33885	559126	PRJNA224116	224116	SAMEA5850799	12519769			10	EMG	na	full-genome-repres	GCA_902373655.1	GCF_902373655.1	identical	425094	1937622

			(firmicutes)															entation					
GCF_005864375.1	ASM586437v1	1254	Pediococcus acidilactici (firmicutes)	1254	Pediococcus acidilactici	Contig	PRJNA543085	543085	PRJNA224116	224116	SAMN11653951	11653951	strain	FAM 18969	60	Agroscope	na	full-genome-representation	GCA_005864375.1	GCF_005864375.1	identical	413925	413925
GCF_900163535.1	ASM90016353v1	1254	Pediococcus acidilactici (firmicutes)	1254	Pediococcus acidilactici	Contig	PRJEB19183	374431	PRJNA224116	224116	SAMEA80353168	6358802	strain	S7	448	MICALIS	na	full-genome-representation	GCA_900163535.1	GCF_900163535.1	identical	405883	405883
GCF_001436795.1	ASM143679v1	1254	Pediococcus acidilactici (firmicutes)	1254	Pediococcus acidilactici	Contig	PRJNA222257	222257	PRJNA224116	224116	SAMN02797809	2797809	strain	AS1.2696	100	Shanghai Majorbio	na	full-genome-representation	GCA_001436795.1	GCF_001436795.1	identical	405421	405421
GCF_011800215.1	ASM1180021v1	1683697	Pediococcus sp. EKM201D (firmicutes)	1683697	Pediococcus sp. EKM201D	Scaffold	PRJNA605474	605474	PRJNA224116	224116	SAMN14070316	14070316	strain	EKM 201D	227	University of Guelph	na	full-genome-representation	GCA_011800215.1	GCF_011800215.1	identical	401933	440959
GCF_011009615.1	ASM1100961v1	1255	Pediococcus pentosaceus (firmicutes)	1255	Pediococcus pentosaceus	Contig	PRJNA517196	517196	PRJNA224116	224116	SAMN10822534	10822534	strain	B4	15	University of Surrey	na	full-genome-representation	GCA_011009615.1	GCF_011009615.1	identical	396170	396170
GCF_000235805.1	ASM23580v2	1080365	Pediococcus acidilactici MA18/5M (firmicutes)	1254	Pediococcus acidilactici	Contig	PRJNA72773	72773	PRJNA224116	224116	SAMN02469945	2469945	strain	MA18/5M	33	Institut Rosell Lallemand Inc.	na	full-genome-representation	GCA_000235805.2	GCF_000235805.1	identical	395998	395998
GCF_009808645.1	ASM980864v1	1255	Pediococcus pentosaceus (firmicutes)	1255	Pediococcus pentosaceus	Scaffold	PRJNA576774	576774	PRJNA224116	224116	SAMN13008395	13008395	strain	FAM 18321	15	Agroscope	na	full-genome-representation	GCA_009808645.1	GCF_009808645.1	identical	389150	389150
GCF_004353295.1	ASM435329v1	1255	Pediococcus pentosaceus (firmicutes)	1255	Pediococcus pentosaceus	Contig	PRJNA525615	525615	PRJNA224116	224116	SAMN11054835	11054835	strain	MZF16	109	University of Rouen Normandie	na	full-genome-representation	GCA_004353295.1	GCF_004353295.1	identical	364301	364301

GCF_009809665.1	ASM980966v1	1255	Pediococcus pentosaceus (firmicutes)	1255	Pediococcus pentosaceus	Contig	PRJNA576774	576774	PRJNA224116	224116	SAMN13008343	13008343	strain	FAM 13073	15	Agroscope	na	full-genome-representation	GCA_009809665.1	GCF_009809665.1	identical	335313	335313
GCF_002553675.1	ASM255367v1	1254	Pediococcus acidilactici (firmicutes)	1254	Pediococcus acidilactici	Contig	PRJNA412075	412075	PRJNA224116	224116	SAMN07702371	7702371	strain	UMN PBX20	50	University of Minnesota	na	full-genome-representation	GCA_002553675.1	GCF_002553675.1	identical	326728	326728
GCF_005864405.1	ASM586440v1	1255	Pediococcus pentosaceus (firmicutes)	1255	Pediococcus pentosaceus	Scaffold	PRJNA543085	543085	PRJNA224116	224116	SAMN11653952	11653952	strain	FAM 19144	23	Agroscope	na	full-genome-representation	GCA_005864405.1	GCF_005864405.1	identical	325547	325547
GCF_011009695.1	ASM1100969v1	1254	Pediococcus acidilactici (firmicutes)	1254	Pediococcus acidilactici	Contig	PRJNA517196	517196	PRJNA224116	224116	SAMN10822530	10822530	strain	E24	15	University of Surrey	na	full-genome-representation	GCA_011009695.1	GCF_011009695.1	identical	318866	318866
GCF_011009715.1	ASM1100971v1	1254	Pediococcus acidilactici (firmicutes)	1254	Pediococcus acidilactici	Contig	PRJNA517196	517196	PRJNA224116	224116	SAMN10822531	10822531	strain	F7	15	University of Surrey	na	full-genome-representation	GCA_011009715.1	GCF_011009715.1	identical	318866	318866
GCF_004099675.1	ASM409967v1	1254	Pediococcus acidilactici (firmicutes)	1254	Pediococcus acidilactici	Scaffold	PRJNA515210	515210	PRJNA224116	224116	SAMN10744163	10744163	strain	L8-A	142	Texas Tech University	na	full-genome-representation	GCA_004099675.1	GCF_004099675.1	identical	315444	315444
GCF_004099815.1	ASM409981v1	1254	Pediococcus acidilactici (firmicutes)	1254	Pediococcus acidilactici	Scaffold	PRJNA515210	515210	PRJNA224116	224116	SAMN10744156	10744156	strain	L2-A	214	Texas Tech University	na	full-genome-representation	GCA_004099815.1	GCF_004099815.1	identical	315282	315282
GCF_009808085.1	ASM980808v1	1255	Pediococcus pentosaceus (firmicutes)	1255	Pediococcus pentosaceus	Contig	PRJNA576774	576774	PRJNA224116	224116	SAMN13008308	13008308	strain	FAM 19169	17	Agroscope	na	full-genome-representation	GCA_009808085.1	GCF_009808085.1	identical	310705	310705
GCF_009808845.1	ASM980884v1	1255	Pediococcus pentosaceus (firmicutes)	1255	Pediococcus pentosaceus	Scaffold	PRJNA576774	576774	PRJNA224116	224116	SAMN13008385	13008385	strain	FAM 18813	16	Agroscope	na	full-genome-representation	GCA_009808845.1	GCF_009808845.1	identical	308715	308715

GCF_009910835.1	ASM991083v1	1254	Pediococcus acidilactici (firmicutes)	1254	Pediococcus acidilactici	Contig	PRJNA544274	544274	PRJNA224116	224116	SAMN11831832	11831832	strain	E1	20	University of Surrey	na	full-genome-representation	GCA_009910835.1	GCF_009910835.1	identical	298407	298407
GCF_011009675.1	ASM1100967v1	1254	Pediococcus acidilactici (firmicutes)	1254	Pediococcus acidilactici	Contig	PRJNA517196	517196	PRJNA224116	224116	SAMN10822532	10822532	strain	I32	15	University of Surrey	na	full-genome-representation	GCA_011009675.1	GCF_011009675.1	identical	298407	298407
GCF_009808815.1	ASM980881v1	1255	Pediococcus pentosaceus (firmicutes)	1255	Pediococcus pentosaceus	Scaffold	PRJNA576774	576774	PRJNA224116	224116	SAMN13008387	13008387	strain	FAM20650	34	Agroscope	na	full-genome-representation	GCA_009808815.1	GCF_009808815.1	identical	297252	297252
GCF_007992275.1	ASM799227v1	1255	Pediococcus pentosaceus (firmicutes)	1255	Pediococcus pentosaceus	Contig	PRJDB6300	400723	PRJNA224116	224116	SAMD00172662	11843515	strain	NBRC107768	141	National Institute of Technology and Evaluation Biological Resource Center	na	full-genome-representation	GCA_007992275.1	GCF_007992275.1	identical	295778	295778
GCF_013385165.1	ASM1338516v1	1255	Pediococcus pentosaceus (firmicutes)	1255	Pediococcus pentosaceus	Contig	PRJNA641973	641973	PRJNA224116	224116	SAMN15374531	15374531	strain	TUCO-3	476	CERELA	na	full-genome-representation	GCA_013385165.1	GCF_013385165.1	identical	295212	295212
GCF_002724015.1	ASM272401v1	1255	Pediococcus pentosaceus (firmicutes)	1255	Pediococcus pentosaceus	Contig	PRJDB6040	396350	PRJNA224116	224116	SAMD00093675	7768543	strain	NBRC3182	107	National Institute of Technology and Evaluation Biological Resource Center	na	full-genome-representation	GCA_002724015.1	GCF_002724015.1	identical	291756	291756
GCF_011800255.1	ASM1180025v1	1683698	Pediococcus sp. EKM202D (firmicutes)	1683698	Pediococcus sp. EKM202D	Scaffold	PRJNA605474	605474	PRJNA224116	224116	SAMN14070325	14070325	strain	EKM202D	235	University of Guelph	na	full-genome-representation	GCA_011800255.1	GCF_011800255.1	identical	287348	440287
GCF_013249135.1	ASM1324913v1	1254	Pediococcus acidilactici (firmicutes)	1254	Pediococcus acidilactici	Contig	PRJNA574342	574342	PRJNA224116	224116	SAMN14848240	14848240	strain	BIO6314	876	Bioprox	na	full-genome-representation	GCA_013249135.1	GCF_013249135.1	identical	270087	270087

GCF_013375165.1	Unicycler	1254	Pediococcus acidilactici (firmicutes)	1254	Pediococcus acidilactici	Scaffold	PRJNA640898	640898	PRJNA224116	224116	SAMN15337232	15337232	strain	IRZ12B	258	Padova university	na	full-genome-representation	GCA_013375165.1	GCF_013375165.1	identical	259848	2024645
GCF_004115445.1	ASM411544v1	1255	Pediococcus pentosaceus (firmicutes)	1255	Pediococcus pentosaceus	Scaffold	PRJNA513828	513828	PRJNA224116	224116	SAMN10721079	10721079	strain	6_1	521	Beijing Technology and Business University	na	full-genome-representation	GCA_004115445.1	GCF_004115445.1	identical	245036	245036
GCF_001672605.1	ASM167260v1	1254	Pediococcus acidilactici (firmicutes)	1254	Pediococcus acidilactici	Contig	PRJNA321943	321943	PRJNA224116	224116	SAMN05162513	5162513	strain	SRCM100320	758	Microbial Institute for Fermentation Industry	na	full-genome-representation	GCA_001672605.1	GCF_001672605.1	identical	230691	230691
GCF_000380265.1	PAD3_1.0	1306952	Pediococcus acidilactici D3 (firmicutes)	1254	Pediococcus acidilactici	Scaffold	PRJNA193440	193440	PRJNA224116	224116	SAMN01991044	1991044	strain	D3	567	Texas A&M University	na	full-genome-representation	GCA_000380265.1	GCF_000380265.1	identical	224342	1865805
GCF_006539025.1	ASM653902v1	1254	Pediococcus acidilactici (firmicutes)	1254	Pediococcus acidilactici	Contig	PRJDB6046	396356	PRJNA224116	224116	SAMD00097159	12056569	strain	NBRC 12231	298	National Institute of Technology and Evaluation Biological Resource Center	na	full-genome-representation	GCA_006539025.1	GCF_006539025.1	identical	212397	212397
GCF_000163095.1	ASM16309v1	563194	Pediococcus acidilactici 7_4 (firmicutes)	1254	Pediococcus acidilactici	Scaffold	PRJNA40051	40051	PRJNA224116	224116	SAMN02463809	2463809	strain	7_4	0	Broad Institute	na	full-genome-representation	GCA_000163095.1	GCF_000163095.1	identical	174286	384055
GCF_007989045.1	ASM798904v1	319652	Pediococcus cellicola (firmicutes)	319652	Pediococcus cellicola	Contig	PRJDB6231	399657	PRJNA224116	224116	SAMD00166038	11282932	strain	NBRC 106103	289	National Institute of Technology and Evaluation Biological Resource Center	na	full-genome-representation	GCA_007989045.1	GCF_007989045.1	identical	160900	160900
GCF_003571945.1	ASM357194v1	1254	Pediococcus acidilactici (firmicutes)	1254	Pediococcus acidilactici	Contig	PRJNA478800	478800	PRJNA224116	224116	SAMN09531784	9531784	strain	GS1	120	Kangwon Natl. Univ.	na	full-genome-representation	GCA_003571945.1	GCF_003571945.1	identical	157835	157835

GCF_004100005.1	ASM410000v1	1254	Pediococcus acidilactici (firmicutes)	1254	Pediococcus acidilactici	Scaffold	PRJNA515210	515210	PRJNA224116	224116	SAMN10744148	10744148	strain	L14-B	67	Texas Tech University	na	full-genome-representation	GCA_004100005.1	GCF_004100005.1	identical	149555	149555
GCF_009809195.1	ASM980919v1	1255	Pediococcus pentosaceus (firmicutes)	1255	Pediococcus pentosaceus	Contig	PRJNA576774	576774	PRJNA224116	224116	SAMN13008369	13008369	strain	FAM 17622	18	Agroscope	na	full-genome-representation	GCA_009809195.1	GCF_009809195.1	identical	131018	131018
GCF_003591195.1	ASM359119v1	1254	Pediococcus acidilactici (firmicutes)	1254	Pediococcus acidilactici	Contig	PRJNA479750	479750	PRJNA224116	224116	SAMN09604260	9604260	strain	WT	160	Kangwon Natl. Univ.	na	full-genome-representation	GCA_003591195.1	GCF_003591195.1	identical	129826	129826
GCF_005864415.1	ASM586441v1	331679	Pediococcus stilesii (firmicutes)	331679	Pediococcus stilesii	Scaffold	PRJNA543085	543085	PRJNA224116	224116	SAMN11653953	11653953	strain	FAM 18815	13	Agroscope	na	full-genome-representation	GCA_005864415.1	GCF_005864415.1	identical	120403	120403
GCF_001593095.1	ASM159309v1	1255	Pediococcus pentosaceus (firmicutes)	1255	Pediococcus pentosaceus	Contig	PRJNA310676	310676	PRJNA224116	224116	SAMN04453291	4453291	strain	FBL2	522	Kyunghee University	na	full-genome-representation	GCA_001593095.1	GCF_001593095.1	identical	117020	117020
GCF_000285875.1	ASM28587v1	1133596	Pediococcus pentosaceus IE-3 (firmicutes)	1255	Pediococcus pentosaceus	Contig	PRJEA80991	80991	PRJNA224116	224116	SAMEA2272526	2484174	strain	IE-3	0	Institute of Microbial Technology (CSIR)	na	full-genome-representation	GCA_000285875.1	GCF_000285875.1	identical	115249	115249
GCF_009809485.1	ASM980948v1	1255	Pediococcus pentosaceus (firmicutes)	1255	Pediococcus pentosaceus	Contig	PRJNA576774	576774	PRJNA224116	224116	SAMN13008353	13008353	strain	FAM 19083	27	Agroscope	na	full-genome-representation	GCA_009809485.1	GCF_009809485.1	identical	102521	102521
GCF_009809425.1	ASM980942v1	1255	Pediococcus pentosaceus (firmicutes)	1255	Pediococcus pentosaceus	Contig	PRJNA576774	576774	PRJNA224116	224116	SAMN13008356	13008356	strain	FAM 19080	21	Agroscope	na	full-genome-representation	GCA_009809425.1	GCF_009809425.1	identical	93775	93775
GCF_009809875.1	ASM980987v1	1255	Pediococcus pentosaceus	1255	Pediococcus pentosaceus	Scaffold	PRJNA576774	576774	PRJNA224116	224116	SAMN13008333	13008333	strain	FAM 19086	15	Agroscope	na	full-genome-repres	GCA_009809875.1	GCF_009809875.1	identical	91657	91657

			(firmicutes)															entation					
GCF_001748065.1	ASM174806v1	1255	Pediococcus pentosaceus (firmicutes)	1255	Pediococcus pentosaceus	Scaffold	PRJDB4140	338436	PRJNA224116	224116	SAMD00036446	5554234	strain	LP28	155	Department of Molecular Biology and Biotechnology, Graduate School of Biomedical and Health Sciences, Hiroshima University	na	full-genome-representation	GCA_001748065.1	GCF_001748065.1	identical	87139	1774865
GCF_001634455.1	ASM163445v1	1254	Pediococcus acidilactici (firmicutes)	1254	Pediococcus acidilactici	Contig	PRJNA308163	308163	PRJNA224116	224116	SAMN04387728	4387728	strain	NRC C1	197	ICAR-National Research Centre on Camel	na	full-genome-representation	GCA_001634455.1	GCF_001634455.1	identical	78213	78213
GCF_001541865.1	ASM154186v1	1255	Pediococcus pentosaceus (firmicutes)	1255	Pediococcus pentosaceus	Contig	PRJNA308373	308373	PRJNA224116	224116	SAMN04395033	4395033	strain	NKY L15	100	Institute of Subtropical Agriculture, Chinese Academy of Sciences	na	full-genome-representation	GCA_001541865.1	GCF_001541865.1	identical	75916	75916
GCF_002770665.1	ASM277066v1	51663	Pediococcus damnosus (firmicutes)	51663	Pediococcus damnosus	Scaffold	PRJNA354877	354877	PRJNA224116	224116	SAMN06053758	6053758	strain	VTT E-123212	50	University of Saskatchewan	na	full-genome-representation	GCA_002770665.1	GCF_002770665.1	identical	74067	2024786
GCF_001641385.1	ASM164138v1	114090	Pediococcus inopinatus (firmicutes)	114090	Pediococcus inopinatus	Contig	PRJDB2940	317444	PRJNA224116	224116	SAMD00018420	4606822	strain	Wikim15	552	World Institute of Kimchi	na	full-genome-representation	GCA_001641385.1	GCF_001641385.1	identical	69828	69828
GCF_001638245.1	ASM163824v1	1254	Pediococcus acidilactici (firmicutes)	1254	Pediococcus acidilactici	Contig	PRJNA309020	309020	PRJNA224116	224116	SAMN04419358	4419358	strain	NRC C3	20	ICAR-National Research Centre on Camel	na	full-genome-representation	GCA_001638245.1	GCF_001638245.1	identical	58553	58553
GCF_007991575.1	ASM799157v1	319653	Pediococcus ethanolidurans	319653	Pediococcus ethanolidurans	Contig	PRJDB6232	399658	PRJNA224116	224116	SAMD00170759	11603097	strain	NBRC 106104	108	National Institute of Technology and Evaluation	na	full-genome-repres	GCA_007991575.1	GCF_007991575.1	identical	56913	56913

			(firmicutes)													Biological Resource Center		entation					
GCF_001461015.1	ASM146101v1	1254	Pediococcus acidilactici (firmicutes)	1254	Pediococcus acidilactici	Contig	PRJNA296933	296933	PRJNA224116	224116	SAMN04111307	4111307	strain	S1	531	Shin's Lab., Kyungpook National University	na	full-genome-representation	GCA_001461015.1	GCF_001461015.1	identical	55717	55717
GCF_001640785.1	ASM164078v1	54062	Pediococcus parvulus (firmicutes)	54062	Pediococcus parvulus	Contig	PRJNA320830	320830	PRJNA224116	224116	SAMN04958102	4958102	strain	2.6	1000	Consiglio per la ricerca in agricoltura e l'analisi dell'economia agraria (CREA)	na	full-genome-representation	GCA_001640785.1	GCF_001640785.1	identical	51115	51115
GCF_002770635.1	ASM277063v1	51663	Pediococcus damnosus (firmicutes)	51663	Pediococcus damnosus	Scaffold	PRJNA354877	354877	PRJNA224116	224116	SAMN06053759	6053759	strain	VTT E-123216	50	University of Saskatchewan	na	full-genome-representation	GCA_002770635.1	GCF_002770635.1	identical	43620	2086833
GCF_002787675.1	ASM278767v1	51663	Pediococcus damnosus (firmicutes)	51663	Pediococcus damnosus	Scaffold	PRJNA354877	354877	PRJNA224116	224116	SAMN06053757	6053757	strain	P58	30	University of Saskatchewan	na	full-genome-representation	GCA_002787675.1	GCF_002787675.1	identical	43387	2087995
GCF_007992295.1	ASM799229v1	480391	Pediococcus argentinicus (firmicutes)	480391	Pediococcus argentinicus	Contig	PRJDB6301	400724	PRJNA224116	224116	SAMD00172663	11843516	strain	NBRC 107827	137	National Institute of Technology and Evaluation Biological Resource Center	na	full-genome-representation	GCA_007992295.1	GCF_007992295.1	identical	42089	42089
GCF_001634445.1	ASM163444v1	1254	Pediococcus acidilactici (firmicutes)	1254	Pediococcus acidilactici	Contig	PRJNA308165	308165	PRJNA224116	224116	SAMN04387731	4387731	strain	NRC C2	53.4	ICAR-National Research Centre on Camel	na	full-genome-representation	GCA_001634445.1	GCF_001634445.1	identical	26857	26857
GCF_000962875.1	ASM96287v1	1448143	Pediococcus damnosus LMG 28219 (firmicutes)	51663	Pediococcus damnosus	Contig	PRJNA234374	234374	PRJNA224116	224116	SAMN02592738	2592738	strain	LMG 28219	270	Ghent University	na	full-genome-representation	GCA_000962875.1	GCF_000962875.1	identical	24659	24659

GCF_006538785.1	ASM653878v1	51663	Pediococcus damnosus (firmicutes)	51663	Pediococcus damnosus	Contig	PRJDB6044	396354	PRJNA224116	224116	SAMD00093694	12056557	strain	NBRC 3889	91	National Institute of Technology and Evaluation Biological Resource Center	na	full-genome-representation	GCA_006538785.1	GCF_006538785.1	identical	20348	20348
GCF_004022295.1	ASM402229v1	1254	Pediococcus acidilactici (firmicutes)	1254	Pediococcus acidilactici	Scaffold	PRJNA512885	512885	PRJNA224116	224116	SAMN10690227	10690227	strain	LPBC 161	11	UFPR	na	full-genome-representation	GCA_004022295.1	GCF_004022295.1	identical	18140	19488
GCF_011009585.1	ASM1100958v1	1254	Pediococcus acidilactici (firmicutes)	1254	Pediococcus acidilactici	Contig	PRJNA517196	517196	PRJNA224116	224116	SAMN10822533	10822533	strain	M16	5	University of Surrey	na	full-genome-representation	GCA_011009585.1	GCF_011009585.1	identical	11317	11317
GCF_001636135.1	ASM163613v1	51663	Pediococcus damnosus (firmicutes)	51663	Pediococcus damnosus	Scaffold	PRJNA309620	309620	PRJNA224116	224116	SAMN04437152	4437152	strain	BM-PD14610	400	South China University of Technology	na	full-genome-representation	GCA_001636135.1	GCF_001636135.1	identical	8404	12875
GCF_001868705.1	ASM186870v1	1254	Pediococcus acidilactici (firmicutes)	1254	Pediococcus acidilactici	Contig	PRJNA344012	344012	PRJNA224116	224116	SAMN05804765	5804765	strain	JKY18	788	Anhui University	na	excluded-from-refseq	GCA_001868705.1	GCF_001868705.1	identical	1643836	1643836
GCA_009761085.1	ASM976108v1	1254	Pediococcus acidilactici (firmicutes)	1254	Pediococcus acidilactici	Contig	PRJNA434783	434783			SAMN08574375	8574375	strain	NCDC 252	30	KURUKSHETRA UNIVERSITY KURUKSHETRA	na	excluded-from-refseq	GCA_009761085.1			358821	358821
GCF_001294765.1	PedAciK3	1254	Pediococcus acidilactici (firmicutes)	1254	Pediococcus acidilactici	Contig	PRJNA293918	293918	PRJNA224116	224116	SAMN04011952	4011952	strain	K3	385	Shin's Lab., Kyungpook National University	na	excluded-from-refseq	GCA_001294765.1	GCF_001294765.1	identical	78870	78870
GCF_001867265.1	ASM186726v1	1254	Pediococcus acidilactici (firmicutes)	1254	Pediococcus acidilactici	Contig	PRJNA336459	336459	PRJNA224116	224116	SAMN05510866	5510866	strain	NRC C4	12	ICAR-National Research Centre on Camel	na	excluded-from-refseq	GCA_001867265.1	GCF_001867265.1	identical	9323	9323
