# Supplementary figures and images for: Genomic Insight into Pediococcus acidilactici HN9, a Potential Probiotic Strain Isolated from the Traditional Thai-Style Fermented Beef Nhang
Source: Microorganisms. 2020 Dec 27;9(1):50. doi: 10.3390/microorganisms9010050 (PMC7823806; doi:10.3390/microorganisms9010050)

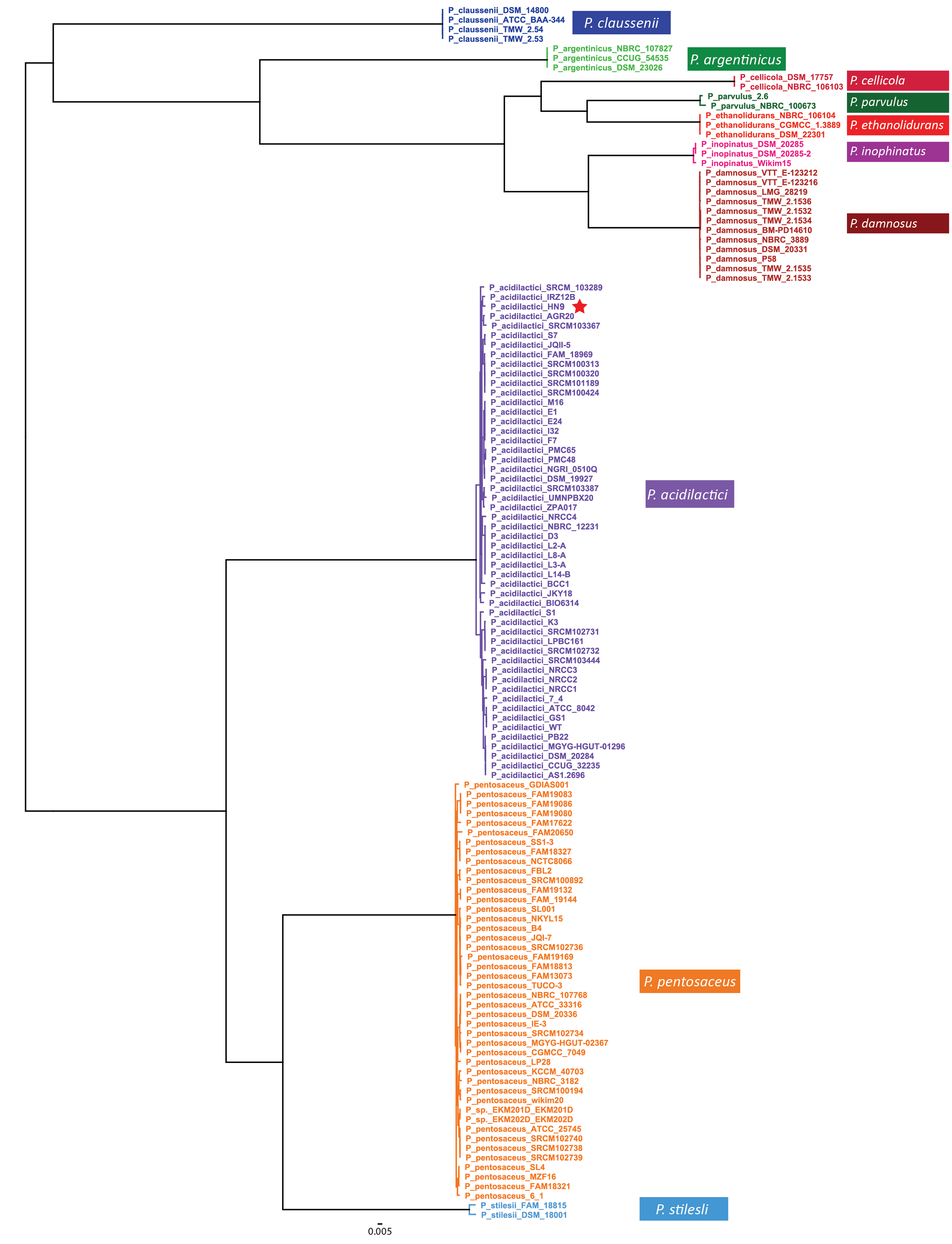

Supplement: Supplementary file 1 [file microorganisms-09-00050-s001.zip › Supplementary/Figure S2.png]

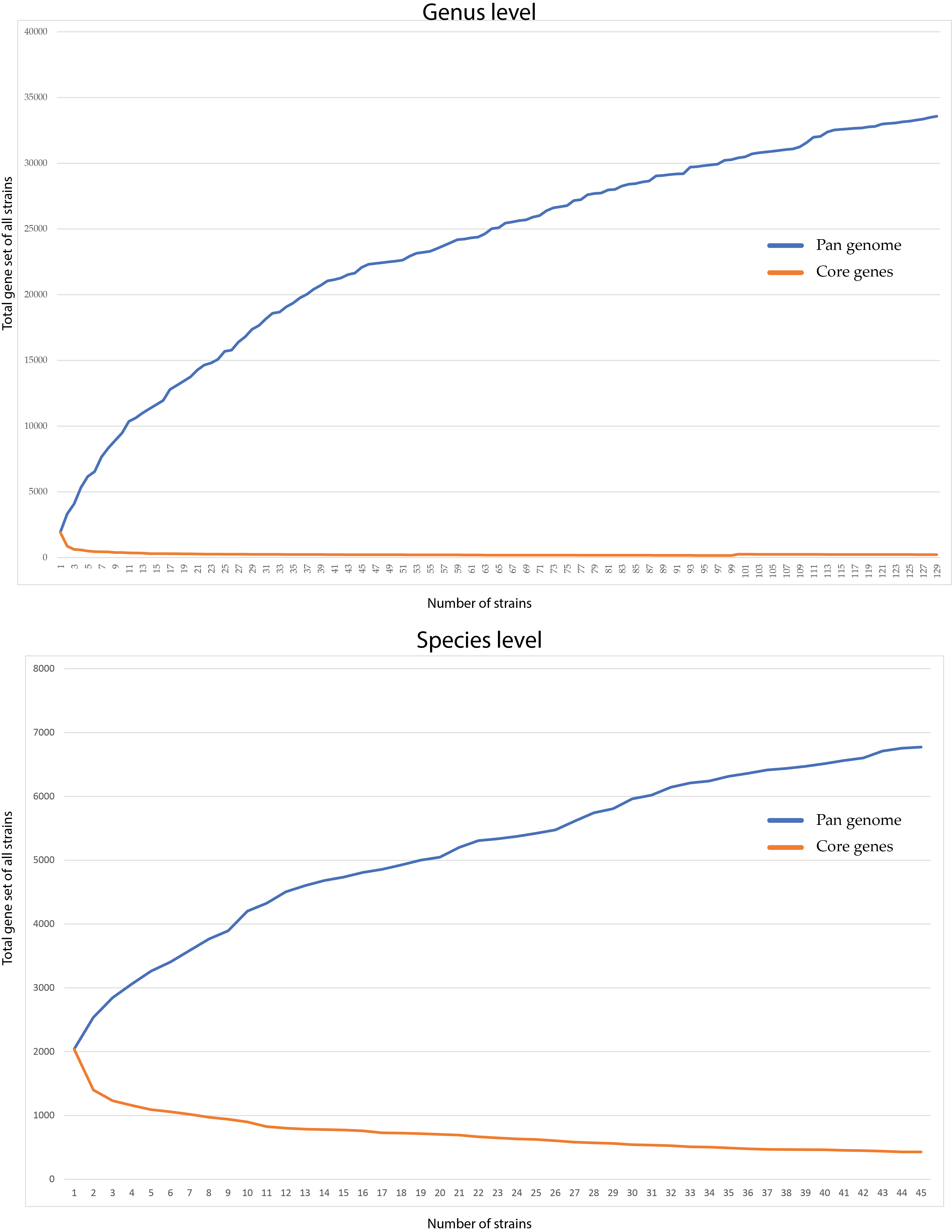

Supplement: Supplementary file 1 [file microorganisms-09-00050-s001.zip › Supplementary/Figure S6.png]

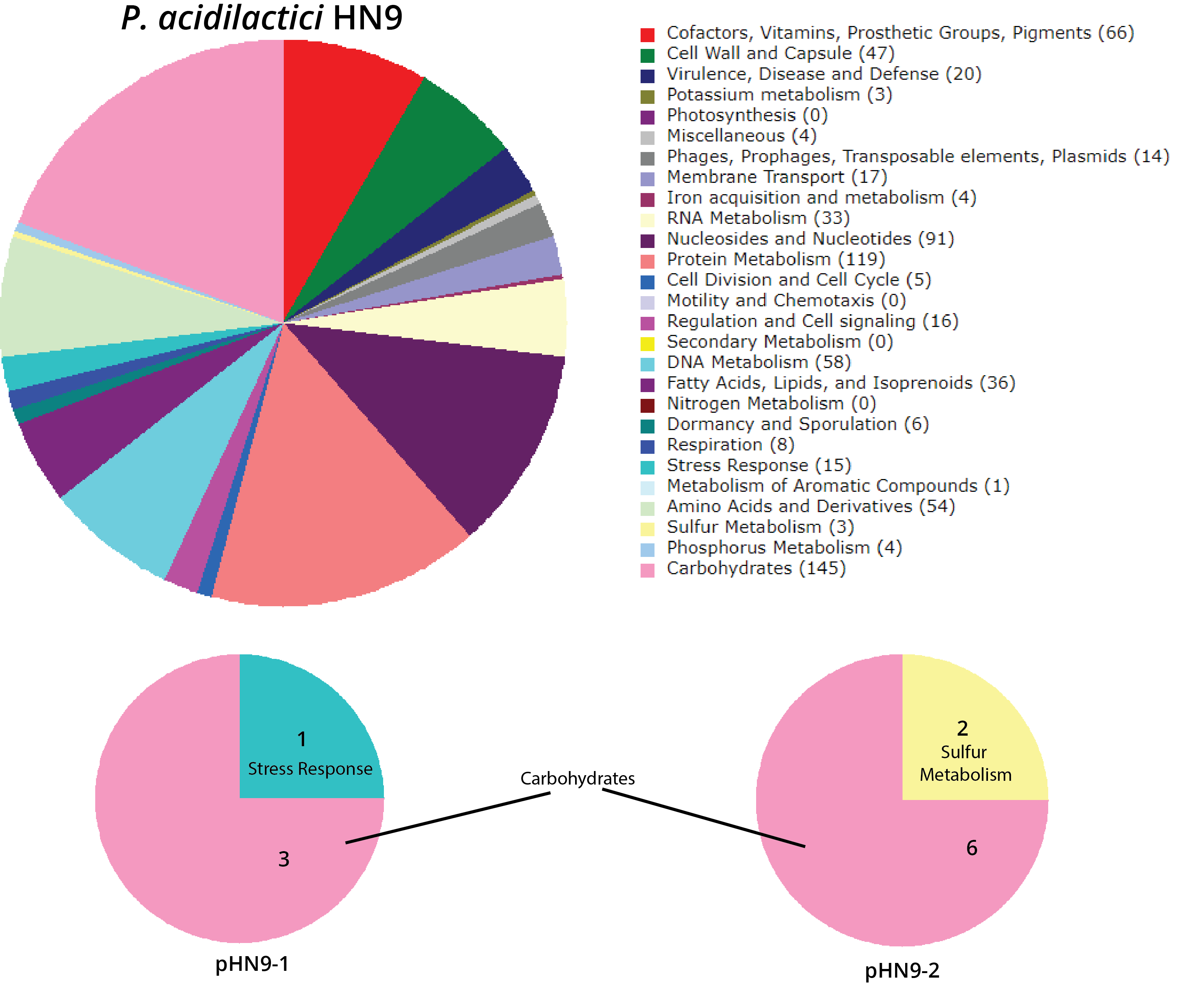

Supplement: Supplementary file 1 [file microorganisms-09-00050-s001.zip › Supplementary/FigureS1.png]

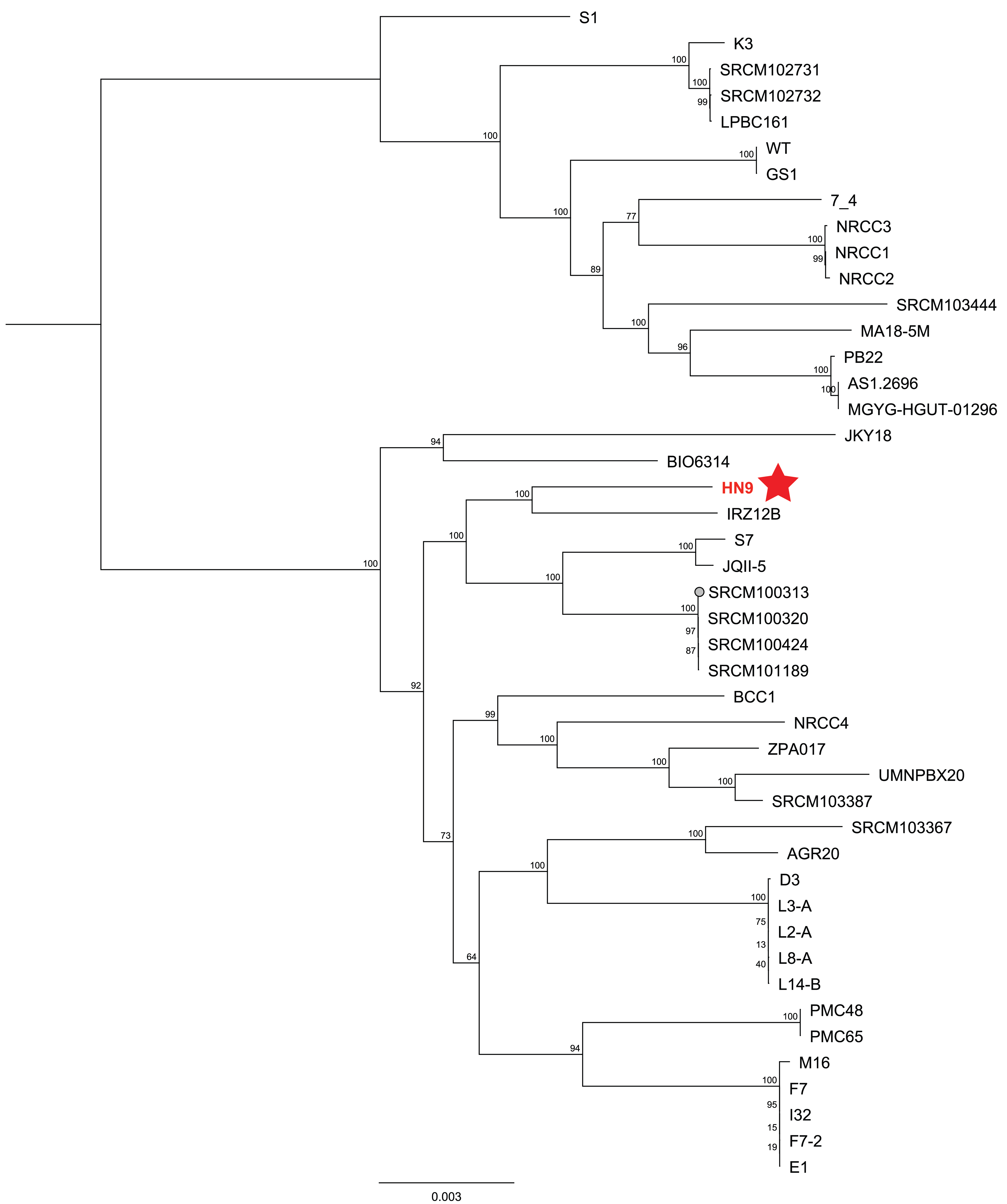

Supplement: Supplementary file 1 [file microorganisms-09-00050-s001.zip › Supplementary/FigureS3.png]

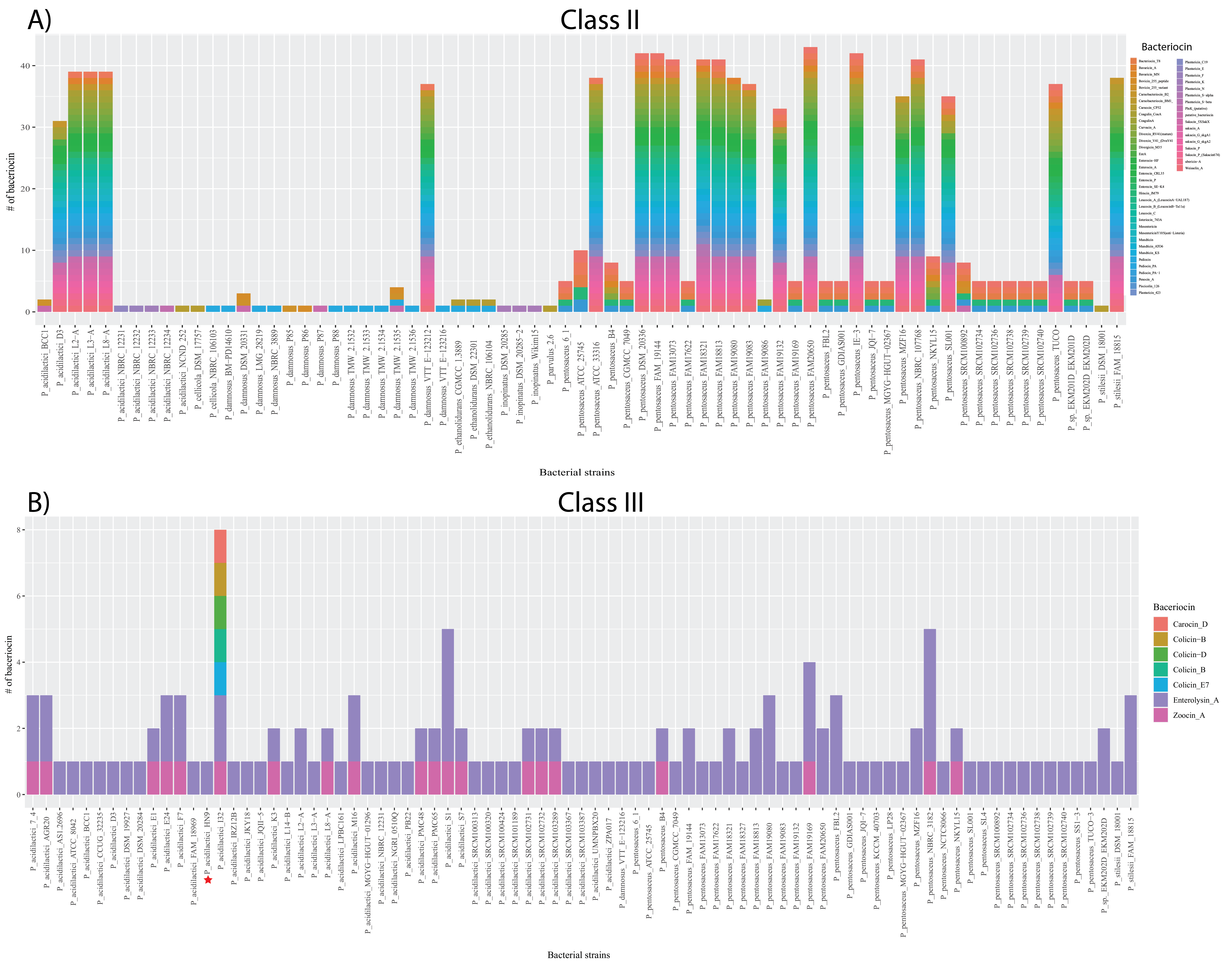

Supplement: Supplementary file 1 [file microorganisms-09-00050-s001.zip › Supplementary/FigureS5.png]
